# Efficient Chemical Protein Synthesis using Fmoc‐Masked N‐Terminal Cysteine in Peptide Thioester Segments

**DOI:** 10.1002/anie.202000491

**Published:** 2020-05-26

**Authors:** Abhisek Kar, Jamsad Mannuthodikayil, Sameer Singh, Anamika Biswas, Puneet Dubey, Amit Das, Kalyaneswar Mandal

**Affiliations:** ^1^ TIFR Centre for Interdisciplinary Sciences Tata Institute of Fundamental Research Hyderabad 36/p Gopanpally Hyderabad 500046 Telangana India; ^2^ Protein Crystallography Section, Radiation Biology and Health Sciences Division Bhabha Atomic Research Centre Trombay Mumbai 400085 India; ^3^ Homi Bhabha National Institute Anushaktinagar Mumbai 400094 India

**Keywords:** chemical protein synthesis, human lysozyme, native chemical ligation, one-pot synthesis, protecting groups

## Abstract

We report an operationally simple method to facilitate chemical protein synthesis by fully convergent and one‐pot native chemical ligations utilizing the fluorenylmethyloxycarbonyl (Fmoc) moiety as an N‐masking group of the N‐terminal cysteine of the middle peptide thioester segment(s). The Fmoc group is stable to the harsh oxidative conditions frequently used to generate peptide thioesters from peptide hydrazide or *o*‐aminoanilide. The ready availability of Fmoc‐Cys(Trt)‐OH, which is routinely used in Fmoc solid‐phase peptide synthesis, where the Fmoc group is pre‐installed on cysteine residue, minimizes additional steps required for the temporary protection of the N‐terminal cysteinyl peptides. The Fmoc group is readily removed after ligation by short exposure (<7 min) to 20 % piperidine at pH 11 in aqueous conditions at room temperature. Subsequent native chemical ligation reactions can be performed in presence of piperidine in the same solution at pH 7.

## Graphical abstract

**All in one**: Highly efficient multisegment native chemical ligation (NCL) has been developed by exploiting the Fmoc group to temporarily mask the N‐terminal Cys of the peptide thioester fragments. Fmoc removal and NCL are achieved in the same reaction mixture through pH adjustments in presence of 20 % piperidine. The simplicity and inherent robustness of this method make it an attractive approach for the high‐yielding total synthesis of proteins.

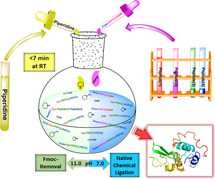

## Introduction

Native chemical ligation (NCL)^[1]^ of unprotected peptide segments made by solid‐phase peptide syntheses (SPPS)^[2]^ has enabled chemical access to a variety of functional protein molecules. The typical size of a functional domain of proteins ranges from 100–200 amino acid residues. Chemical syntheses of such large protein domains can only be realized through multisegment native chemical ligation reactions.^[3]^ However, the traditional multisegment ligation strategies are associated with multiple purifications and freeze‐drying sequences, which are time‐consuming, laborious, and low‐yielding due to handling losses in intermediate purification steps. In this regard, an important step forward in the field of chemical protein synthesis has been one‐pot native chemical ligation, where multiple peptide segments are ligated, sequentially, without intermediate purifications.^[4]^

Another major advance in the field has been stimulated by the introduction of fully convergent chemical protein synthesis using kinetically controlled ligation^[5a]^ or peptide‐hydrazide chemistry.^[5b]^ In convergent synthesis, two halves of the polypeptide chain, synthesized independently in parallel from two or more peptide segments using NCL, are joined in a final step to give the full‐length polypeptide chain. Fully convergent chemical syntheses are always efficient in terms of purity and yield;^[6]^ and have been widely applied for the synthesis of number of large protein molecules.^[5]^

**Figure 1:**
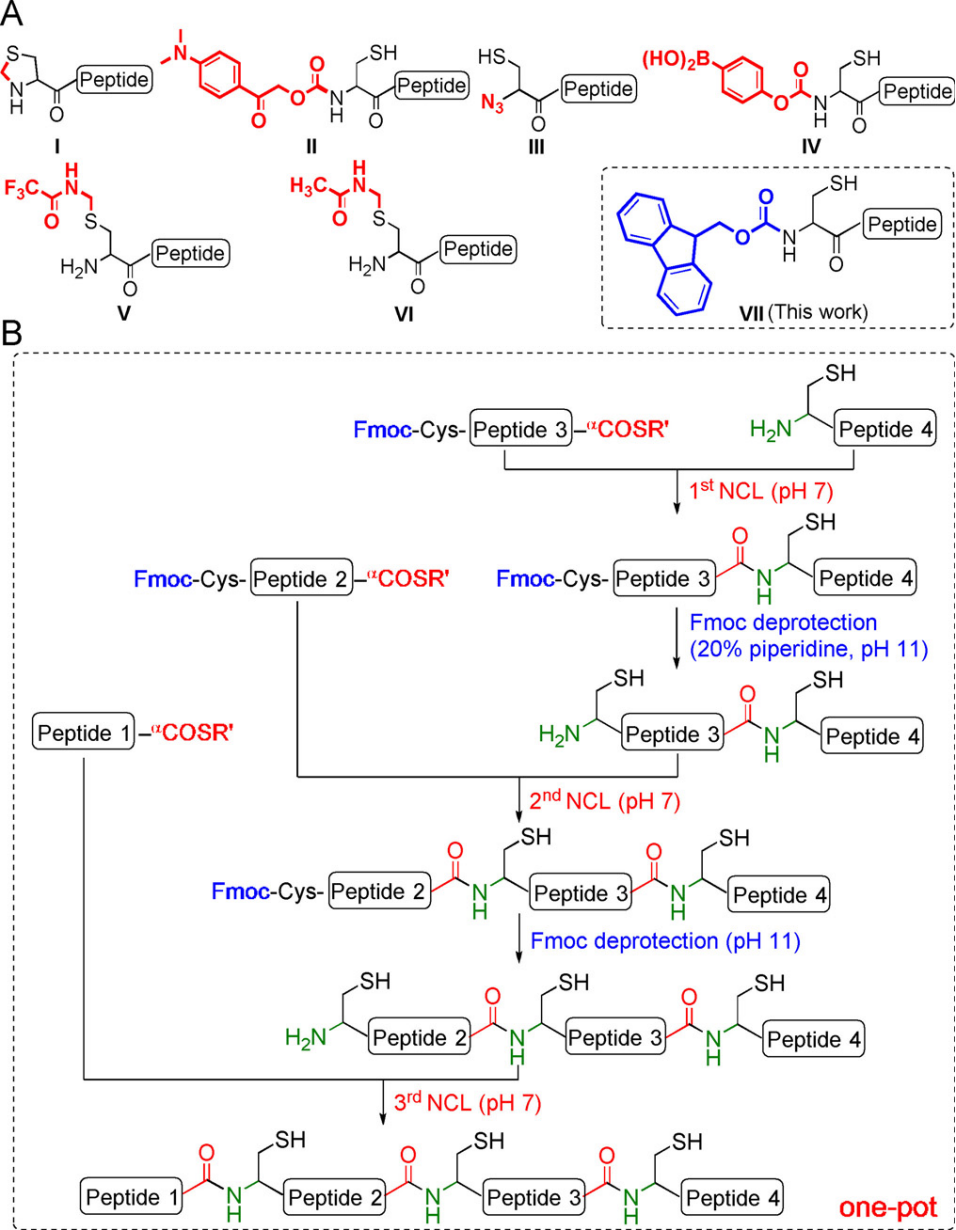
A) Protection of the N‐terminal Cys residue: **I**,^[4a]^ **II**,^[4d]^ **III**,^[4e]^ **IV**,^[4f]^ **V**,^[4g]^ **VI** ^[4h]^ are previously reported and **VII** is this work, for one‐pot NCL. B) Schematic representation of multisegment one‐pot NCL using Fmoc as the temporary masking group of the reactive N‐terminal Cys residue.

Both one‐pot multisegment ligation from the C‐terminal peptide segment towards the N‐terminal segment and fully convergent synthesis mandate temporary protection of the reactive cysteine residue located at the N‐terminus of middle peptide thioester segment(s). In the first demonstration of one‐pot NCL, Bang et al. utilized (4R)‐1,3‐thiazolidine carboxylic acid (Thz) as a cryptic form of the N‐terminal Cys residue of the middle peptide thioester segment prepared by Boc‐chemistry SPPS.^[4a]^ The same strategy was used for the synthesis of several other moderately sized protein molecules, where peptide thioester syntheses were performed using Boc‐chemistry SPPS.[^4b^, ^4c^] Liu and co‐workers introduced peptide hydriazides^[7a]^ and Dawson's group introduced peptide *o*‐aminoanilides[^7b^, ^7c^, ^7d^] as surrogates for peptide thioester segments used in chemical protein synthesis. Because peptide hydrazides or *o*‐aminoanilides are readily prepared by Fmoc‐chemistry SPPS, they are widely used. However, the thiazolidine group was found to be incompatible with the oxidative NaNO_2_ treatment necessary for activation of the peptide thioester surrogates, peptide *o*‐aminoanilides, and peptide hydrazides^[7e]^ prepared by the more widely used Fmoc‐chemistry SPPS. This observation compelled researchers to develop alternative chemical tactics for one‐pot ligations and convergent syntheses compatible with the peptide *o*‐aminoanilides[^5e^, ^7c^] and peptide hydrazides.[^4d^, ^4e^, ^4f^, ^4g^, ^4h^, ^5b^] Although several alternative cysteine protecting groups (Figure 1 A) have been reported for these applications, none have found widespread use because they require either multistep synthetic route to protect cysteine or careful chemical manipulations to prevent side reactions during deprotection of the cysteine in aqueous solution.[^4d^, ^4e^, ^4f^, ^4g^, ^4h^]

Herein, we report the use of the fluorenylmethyloxycarbonyl (Fmoc) group,^[8]^ pre‐installed on cysteine for routine Fmoc‐chemistry SPPS, as an operationally simple and robust method for masking the N‐terminal reactive cysteine residue of peptide (Cys–peptide) thioester segments. We show that the Fmoc group is stable to NaNO_2_ treatment and can be removed quantitatively by a brief exposure to 20 % piperidine in aqueous ligation buffer. Moreover, the presence of piperidine in the ligation buffer does not interfere with the subsequent ligation reaction at neutral pH, thereby enabling multisegment peptide ligations in a one‐pot manner. Clean conversion in every synthetic step gives high‐purity full‐length polypeptide in excellent overall yields.

## Results and Discussion

**Figure 2:**
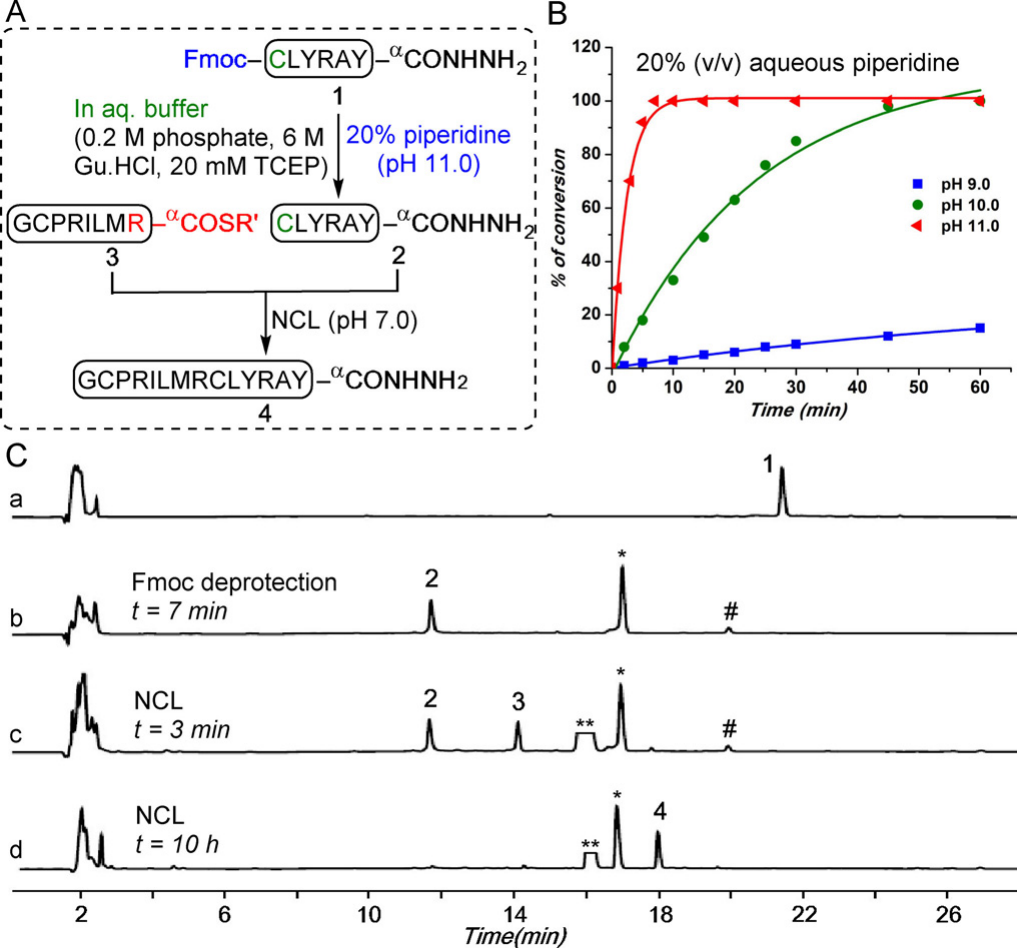
Fmoc removal and NCL reaction in the same reaction mixture. A) Synthetic strategy for Fmoc removal from model peptide **1** and NCL of the resulting Cys–peptide **2** and peptide thioester **3** in the same reaction solution. R′= CH_2_CH_2_SO_3_Na. B) Fmoc‐removal kinetics in the presence of 20 % piperidine in aqueous ligation buffer containing 200 mm phosphate, 6 m Gu.HCl, 20 mm TCEP at pH 9 (blue ▪), 10 (green •) and 11 (red ▴). Solid lines represent the fitted plot over experimental data points. C) Analytical HPLC profiles (*λ*=214 nm) for: a) Purified peptide **1** in standard ligation buffer (200 mm phosphate, 6 m Gu.HCl, 20 mm TCEP); b) Fmoc removal in presence of 20 % (v/v) piperidine in ligation buffer to give **2** within 7 min. * represents dibenzofulvene‐TCEP adduct and **#** represents dibenzofulvene‐piperidine adduct (see Section S3 in the Supporting Information); c) 3 min after addition of peptide **3** and MPAA. ** indicates MPAA; d) After completion of the ligation reaction to afford ligated product **4**.

The success of the proposed one‐pot synthetic strategy (Figure 1 B) relied primarily on the efficient removal of the Fmoc group in aqueous buffer. We found, using the model peptide Fmoc‐Cys–Leu–Tyr–Arg–Ala–Tyr‐^α^CONHNH_2_ (**1**) where the Fmoc group of the N‐terminal Cys residue was left intact (Figure 2 A), that 20 % (v/v) aqueous piperidine at pH 11 was optimal for the quantitative deprotection of the Fmoc group within 7 minutes (Figure 2 B; for optimization details see Section S3 in the Supporting Information).

### Fmoc Removal and NCL in the Same Reaction Mixture

In order to evaluate the feasibility of Fmoc removal combined with native chemical ligation in the same reaction mixture, we ligated a model thioester peptide Gly–Cys–Pro–Arg–Ile–Leu–Met–Arg‐^α^COSCH_2_CH_2_SO_3_Na (**3**) with the model Cys–peptide hydrazide Cys–Leu–Tyr–Arg–Ala–Tyr‐^*α*^CONHNH_2_ (**2**) in standard ligation buffer (200 mm phosphate, 6 m Gu.HCl, 20 mm TCEP), as shown in Figure 2 A. The Cys–peptide hydrazide **2** was obtained by Fmoc removal on peptide **1** using 20 % piperidine in ligation buffer at pH 11.0. After Fmoc removal, the subsequent NCL with the thioester peptide **3** was carried out in the same solution at neutral pH in the presence of exogenous aryl thiol catalyst (20 mm 4‐mercaptophenylacetic acid (MPAA)) and resulted in formation of the desired ligated product **4**, without formation of any undesired side products (Figure 2 C). The presence of 20 % piperidine in the reaction mixture did not interfere with the native chemical ligation. Since the pKa of piperidine is 11.1, presumably the piperidine remained protonated at neutral pH, thereby preventing nucleophilic attack on the thioester.

### Compatibility of Asn–Gly and Asp–Gly Sequences with Piperidine Treatment

The presence of Asn–Gly or Asp–Gly sequences makes any peptide vulnerable to deamidation or iso‐aspartic acid formation through the intermediacy of aspartimide at high pH, such as the pH 11 used to remove the Fmoc group in our method.^[9]^ Therefore, it was imperative to check the compatibility of Asn–Gly or Asp–Gly sequences with the Fmoc removal conditions used in our study. In order to assess the stability of the Asn–Gly or Asp–Gly peptides, we incubated two model peptides containing Asn–Gly or Asp–Gly in their sequence at pH 11 in presence of 20 % piperidine. We found no detectable iso‐aspartyl peptide formation from the Asp–Gly sequence and a negligible amount of deamidated product and iso‐aspartyl peptide formation from the Asn–Gly sequence upon exposure to the optimized Fmoc removal conditions at pH 11, even up to 14 minutes (see Section S4.1 and Figure S7a—c in the Supporting Information).

### Multisegment Polypeptide Synthesis

**Figure 3:**
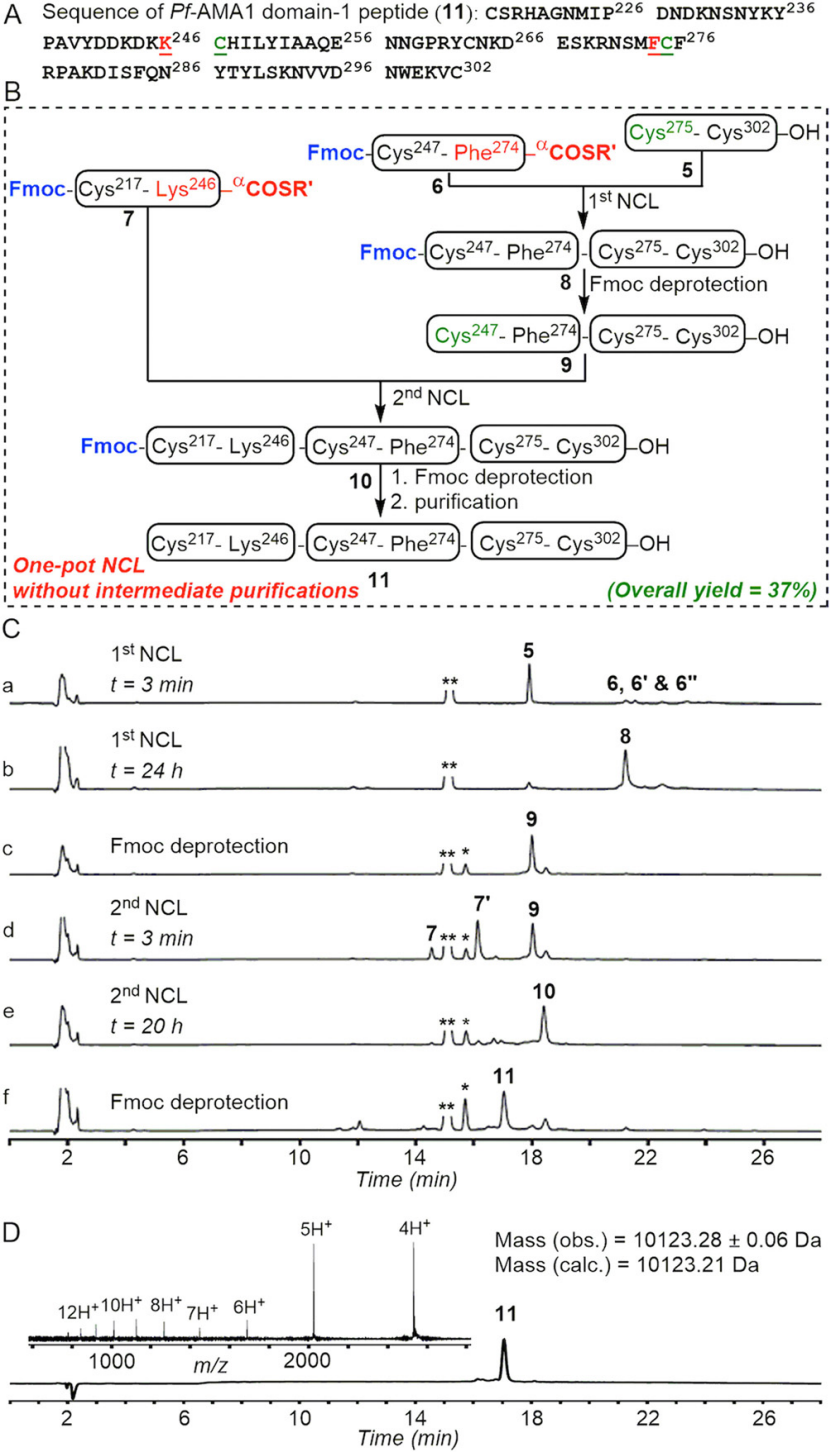
Multisegment polypeptide synthesis with one‐pot ligation sequences. A) Sequence of the polypeptide segment of *P. falciparum* protein *Pf*‐AMA1 (3D7 strain) **11**. NCL sites are underlined and highlighted in red and green. B) One‐pot ligation without any intermediate purification step resulted in the final polypeptide in 37 % overall yield. R′=CH_2_CH_2_SO_3_Na. C) Analytical HPLC monitoring of the one‐pot ligation reaction: a) 3 min after the addition of the peptide Cys^275^–Cys^302^‐COOH (**5**) and Fmoc‐Cys^247^–Phe^274^‐^α^COCH_2_CH_2_SO_3_Na (**6**) in the standard ligation buffer (200 mm PB, 6 m Gu.HCl, 20 mm TCEP) containing 20 mm MPAA. **6*′*** and **6′′** indicate thiolactone formation from **6**; b) The first ligation, within 24 h, resulted in Fmoc‐Cys^247^–Cys^302^‐COOH (**8**) as the ligated product; c) Fmoc removal from the ligated peptide **8** to give peptide Cys^247^–Cys^302^‐COOH (**9**); d) 3 min after the addition of peptide Fmoc‐Cys^217^–Lys^246^‐^α^COCH_2_CH_2_SO_3_Na (**7**) in the reaction mixture; e) The second ligation was essentially complete within 20 h and furnished polypeptide Fmoc‐Cys^217^–Cys^302^‐COOH (**10**); f) Fmoc removal from the ligated product **10** to give target peptide Cys^217^–Cys^302^‐COOH (**11**). * and ** indicate dibenzofulvene‐TECP adduct and MPAA, respectively. D) Analytical HPLC profile (*λ*=214 nm) with ESI‐MS showing charge‐state distribution (inset) of the purified polypeptide **11**; observed mass 10 123.28±0.06 Da (average of the eight most abundant charge states) and calculated mass 10 123.21 Da (average isotope composition).

In order to evaluate the efficacy of consecutive one‐pot NCL reactions using Fmoc‐Cys–peptide thioester segments, we compared a typical synthesis of a larger polypeptide through C‐to‐N one‐pot ligations *without* intermediate purification steps. We selected an 86‐residue polypeptide segment (**11**, Cys^217^–Cys^302^) from *Plasmodium falciparum* protein *Pf*‐AMA1 (3D7 strain), which contains a pH‐sensitive Asn–Gly sequence, as a target for synthesis (Figure 3 A).

For the one‐pot three‐segment C‐to‐N sequential ligation synthesis (Figure 3 B), Fmoc protection of the N‐terminal Cys residue of both the middle segment and the N‐terminal segment was not removed after chain assembly by standard Fmoc‐chemistry SPPS. The corresponding peptide thioesters, Fmoc‐Cys^247^–Phe^274^‐^α^COCH_2_CH_2_SO_3_Na (**6**) and Fmoc‐Cys^217^–Lys^246^‐^α^COCH_2_CH_2_SO_3_Na (**7**), respectively, were obtained by activation of the peptide hydrazides by treatment with NaNO_2_ followed by thiolysis with sodium 2‐mercaptoethane sulfonate (MESNa). The Fmoc group in both peptides remained intact during the NaNO_2_ treatment, and the thioester peptides were obtained in excellent crude purity (see Section S6 in the Supporting Information).

With all three peptide segments in hand, we first ligated the peptide **5** and **6** in ligation buffer (pH 6.9) containing 100 mm MPAA [Figure 3 C, (b)]. The pH of the reaction mixture was then raised to 11 by adding piperidine (20 % (v/v), final concentration) in order to remove the N‐terminal Fmoc group of the ligated product **8**, giving quantitative Fmoc deprotection of **8** within 7 min to yield peptide **9** [Figure 3 C, (c)]. To carry out the subsequent NCL reaction with the N‐terminal peptide segment **7** in one‐pot, the pH of the reaction mixture was brought down to 6.9 [Figure 3 C, (d)]. After completion of the ligation reaction [Figure 3 C, (e)], we again raised the pH of the reaction mixture to 11, maintaining the concentration of piperidine to 20 % (v/v), to remove the Fmoc group from the ligated polypeptide **10** [Figure 3 C, (f)]. After the two ligations and two Fmoc‐removal steps in one‐pot, a single purification was carried out to obtain full‐length polypeptide **11** in 37 % yield based on the limiting peptide **5** (Figure 3 D).

**Figure 4:**
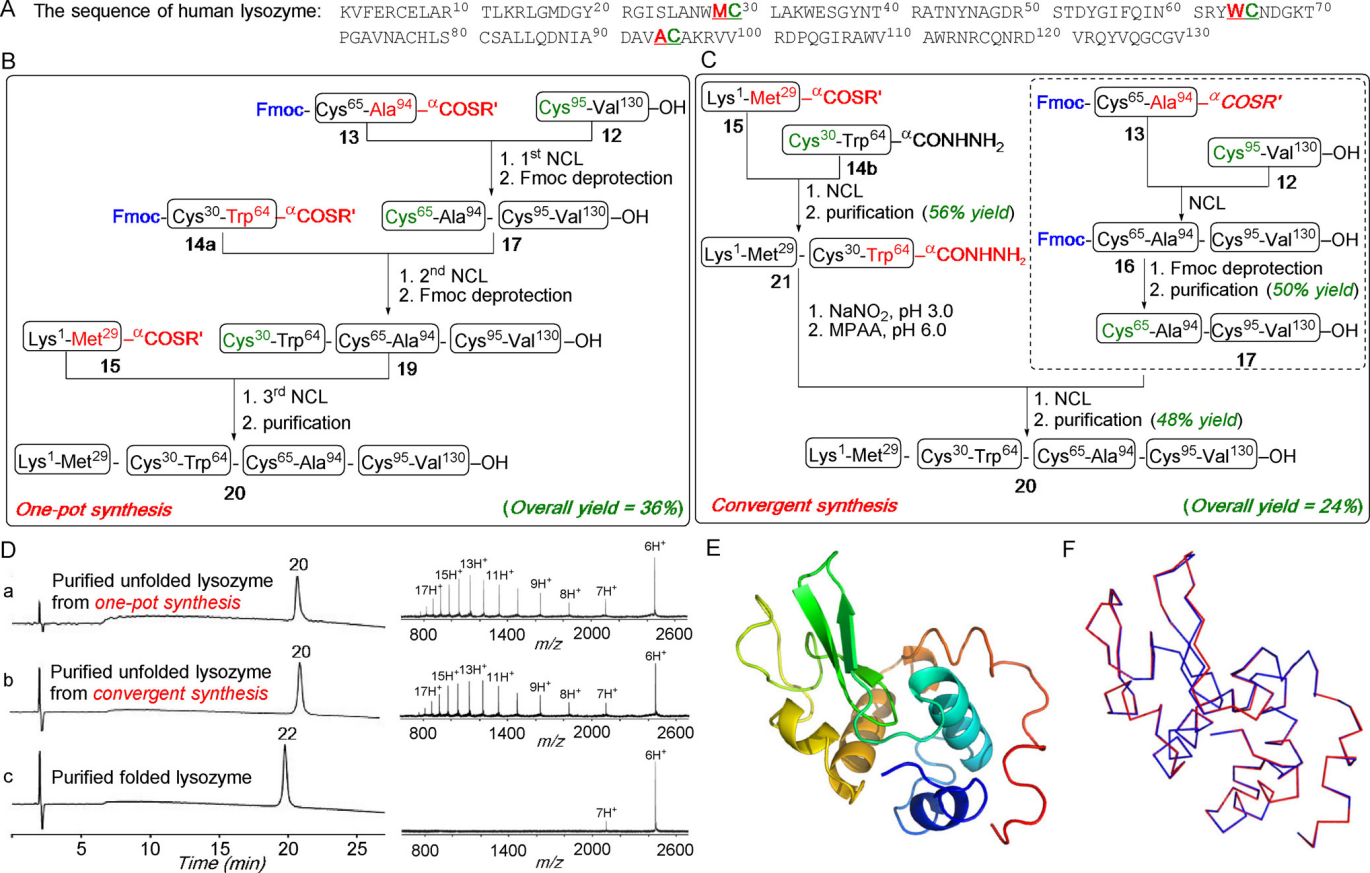
Total chemical synthesis of human lysozyme. A) Amino acid sequence of human lysozyme polypeptide chain [UniProtKB: B2R4C5]. NCL sites are underlined and highlighted in red and green. B) Synthetic strategy for the one‐pot synthesis. C) Synthetic strategy for the convergent synthesis; R′=CH_2_CH_2_SO_3_Na, Ar=C_6_H_4_CH_2_COOH. D) Analytical HPLC chromatogram (left, *λ*=214 nm) together with ESI‐MS data (right) for: a) purified unfolded full‐length lysozyme polypeptide **20** obtained from the one‐pot synthesis [observed mass 14 700.70±0.04 Da (average isotope composition) and calculated mass 14 700.63 Da (average isotope composition)]; b) purified unfolded full‐length lysozyme polypeptide **20** obtained from the convergent synthesis (observed mass 14 700.72±0.03 Da (average isotope composition) and calculated mass 14 700.63 Da (average isotope composition); c) folded human lysozyme **22** [observed mass 14 692.71±0.07 Da (average of the most abundant isotopologue mass over two observed charge states) and calculated mass 14 692.56 Da (average isotope composition)]. Note the earlier retention time and the narrow distribution of the charge states of the folded lysozyme protein compared with the unfolded polypeptide. E) A cartoon representation of the X‐ray crystal structure of the chemically synthesized human lysozyme. F) Figure showing the alignment of our chemically synthesized human lysozyme structure (this work, PDB ID: 6LFH) with the biosynthetic human lysozyme crystal structure at comparable resolution (1.4 Å resolution, PDB ID: 1JWR),^[10f]^ showing an RMSD for C‐α atoms of 0.132 Å.

Having established an efficient method for Fmoc removal and NCL reactions in the same reaction solution, we wanted to explore the utility of this method for the total chemical synthesis of a typical protein. Human lysozyme contains a polypeptide chain of 130 amino acid residues that includes all twenty of the common proteinogenic amino acids in its sequence (Figure 4 A). It has been employed as a model system in various biochemical and biophysical studies directed towards understanding enzyme catalysis, protein folding, and amyloidogenesis.[^10a^, ^10b^, ^10c^] For the total chemical synthesis, we strategically divided the full‐length lysozyme polypeptide into four peptide segments.^[5c]^

### One‐Pot Synthesis of the Human Lysozyme Polypeptide Chain

The one‐pot synthetic strategy is shown in Figure 4 B. Synthesis of the peptide segments is described in Sections S7.1–S7.4 in the Supporting Information. The first NCL reaction between the Cys–peptide **12** and the peptide thioester **13** at pH 6.7 afforded the ligated product, which was then subjected to 20 % (v/v) piperidine treatment at pH 11 to furnish the deprotected peptide **17** within 7 min (see Section S7.5 and Figure S21 in the Supporting Information). We then reduced the pH of the reaction mixture and carried out the second ligation reaction with the peptide segment **14 a** at pH 6.7. The next Fmoc removal cycle in the same reaction mixture, by simply raising the pH to 11.0, maintaining the piperidine concentration to 20 % (v/v), led to complete removal of the Fmoc group from the resultant ligated peptide **18** to afford the deprotected polypeptide **19**. The same pH adjustments were repeated for the final ligation reaction with the peptide thioester segment **15** in one pot to deliver the full‐length lysozyme polypeptide **20**. After five synthetic steps, a single purification was carried out to give full‐length human lysozyme polypeptide **20** in good yield (36 % based on the starting peptide segment **12**). The homogeneity of the synthetic product and its mass were confirmed by LC–MS [Figure 4 D, (a)]. The full‐length polypeptide had an observed mass 14 700.70±0.04 Da (average isotope composition) and calculated mass 14 700.63 Da (average isotope composition).

### Convergent Synthesis of the Human Lysozyme Polypeptide Chain

In principle, fully convergent synthesis has advantages over consecutive synthetic reactions or partially convergent synthesis.^[6]^ In order to demonstrate the application of Fmoc protection of N‐terminal Cys in peptide thioester segments in a fully convergent synthesis, we prepared the same lysozyme polypeptide as depicted in Figure 4 C.

For the convergent synthesis of the lysozyme polypeptide chain, the N‐terminal half of the full‐length polypeptide was prepared by NCL of the Cys‐terminal peptide hydrazide **14 b** and the peptide thioester **15** to afford polypeptide hydrazide **21** (see Figure S23). The C‐terminal half of the full‐length lysozyme polypeptide was obtained, as discussed in the one‐pot strategy, by NCL of Cys–peptide **12** and the Fmoc‐protected peptide thioester **13**, followed by piperidine mediated Fmoc removal to give polypeptide **17** (see Figure S24). Both **17** and **21** were purified by reverse‐phase HPLC, then reacted after conversion of **21** to the thioester by treatment with NaNO_2_ at reduced temperature followed by addition of MPAA, after which Cys–peptide **17** was added to effect the final NCL reaction at pH 6.7 and to afford the full‐length lysozyme polypeptide **20** (see Figure S25) in 48 % yield (based on the starting peptide segment **17**) after purification. The full‐length polypeptide had an observed mass 14 700.72±0.03 Da (average isotope composition) and calculated mass 14 700.63 Da (average isotope composition). The purities of the full‐length lysozyme polypeptide (**20**) products obtained from convergent synthesis and the one‐pot sequential reactions were similar [Figure 4 D, (a,b)] although the overall yield was slightly reduced.

### Folding to Obtain correctly Disulfide‐Linked Human Lysozyme

Finally, the full‐length polypeptide **20** was folded with concomitant formation of disulfide bonds in buffer containing a redox system consisting of 5 mm oxidized glutathione, 2 mm DTT in presence of 0.8 m Gu⋅HCl and 1 mm EDTA in 0.1 m TRIS buffer (pH 8) to furnish folded lysozyme protein **22** after HPLC purification in 38 % yield of isolated product (based on the amount of lysozyme polypeptide **20**).^[5c]^ The folded lysozyme protein had an observed mass of 14 692.71±0.07 Da (average of the most abundant isotopologue mass over two observed charge states), 8.0 Da less than the mass of the full length polypeptide chain (14 700.7 Da), which is in an excellent agreement with the formation of four disulfide bonds with loss of eight protons [Figure 4 D, (c)]. The earlier retention‐time shift compared with the unfolded lysozyme molecule in reverse‐phase HPLC and the altered charge distribution in the ESI‐MS, characteristics of a folded globular protein, was clearly evident for our chemically synthesized lysozyme molecule. To the best of our knowledge this is only the second successful total synthesis of human lysozyme,^[5c]^ after several unsuccessful attempts made in the 1970s from leading laboratories.[^10d^, ^10e^]

To confirm the correct disulfide bonding pattern and the 3D structure of the folded protein molecule, we crystallized the chemically synthesized lysozyme protein using reported^[5c]^ crystallization condition and determined its 3D structure by X‐ray crystallography. The lysozyme crystals were obtained by mixing 2 μL of a solution containing 14 mg mL^−1^ of folded synthetic human lysozyme **22** in 120 mm LiCl, 2.5 mm HEPES, pH 7.5, and 2 μL of reservoir solution consisting of 30 mm sodium phosphate, 2.5 m NaCl, pH 4.9 using hanging drop vapor diffusion method. We used a crystal that diffracted to 1.46 Å resolution to solve the structure by molecular replacement using the coordinates from PDB ID: 2NWD as the search model for phase determination. The crystallographic model was finally refined using Phenix.^[11]^ The refined model had an R*work*/R*free* of 16.2 %/19.6 %. The obtained 3D model (Figure 4 E) revealed correct disulfide formation and had a C‐α root mean square deviation (RMSD) of 0.132 Å (Figure 4 F) compared with the crystal structure reported for recombinant lysozyme protein at a similar resolution.^[10f]^

## Conclusion

In summary, we have developed a robust and operationally simple method for total protein synthesis by multisegment, fully convergent, or one‐pot native chemical ligations using the Fmoc moiety as a temporary masking group of the N‐terminal reactive Cys residue of the peptide thioester segment(s). The Fmoc group was fully compatible with the NaNO_2_ treatment frequently used for the generation of peptide thioesters from peptide hydrazides or peptide *o*‐aminoanilides. Fmoc removal was readily achieved using a short exposure of the peptide to 20 % piperidine in aqueous conditions. The presence of piperidine did not interfere with the subsequent ligation reactions in one‐pot usually carried out at pH 7. Notably, our method provides an alternative and highly efficient approach for high‐yielding chemical protein synthesis.

## Conflict of interest

The authors declare no conflict of interest.

## Supporting information

As a service to our authors and readers, this journal provides supporting information supplied by the authors. Such materials are peer reviewed and may be re‐organized for online delivery, but are not copy‐edited or typeset. Technical support issues arising from supporting information (other than missing files) should be addressed to the authors.

Supplementary

## References

[1] P. E. Dawson , T. W. Muir , I. Clark-Lewis , S. B. Kent , Science 1994, 266, 776–779. 7973629 10.1126/science.7973629

[2a] R. B. Merrifield , J. Am. Chem. Soc. 1963, 85, 2149–2154;

[2b] M. Schnölzer , P. Alewood , A. Jones , D. Alewood , S. B. H. Kent , Int. J. Pept. Res. Ther. 2007, 13, 31–44;

[2c] A. J. Mijalis , D. A. Thoma , M. D. Simon , A. Adamo , R. Beaumont , K. F. Jensen , B. L. Pentelute , Nat. Chem. Biol. 2017, 13, 464–468. 28244989 10.1038/nchembio.2318

[3a] S. B. H. Kent , Protein Sci. 2019, 28, 313–328; 30345579 10.1002/pro.3533 PMC6319755

[3b] S. Kent , Bioorg. Med. Chem. 2017, 25, 4926–4937; 28687227 10.1016/j.bmc.2017.06.020

[3c] V. Agouridas , O. El Mahdi , V. Diemer , M. Cargoet , J. C. M. Monbaliu , O. Melnyk , Chem. Rev. 2019, 119, 7328–7443; 31050890 10.1021/acs.chemrev.8b00712

[3d] J. B. Li , S. Tang , J. S. Zheng , C. L. Tian , L. Liu , Acc. Chem. Res. 2017, 50, 1143–1153; 28374993 10.1021/acs.accounts.7b00001

[3e] S. Bondalapati , M. Jbara , A. Brik , Nat. Chem. 2016, 8, 407–418. 27102674 10.1038/nchem.2476

[4a] D. Bang , S. B. H. Kent , Angew. Chem. Int. Ed. 2004, 43, 2534–2538; 10.1002/anie.200353540 15127445

[4b] D. Bang , G. I. Makhatadze , V. Tereshko , A. A. Kossiakoff , S. B. Kent , Angew. Chem. Int. Ed. 2005, 44, 3852–3856; 10.1002/anie.200463040 15834850

[4c] K. Mandal , S. B. H. Kent , Angew. Chem. Int. Ed. 2011, 50, 8029–8033; 10.1002/anie.201103237 PMC3478761 21744452

[4d] S. Ueda , M. Fujita , H. Tamamura , N. Fujii , A. Otaka , ChemBioChem 2005, 6, 1983–1986; 16206319 10.1002/cbic.200500272

[4e] M. Pan , Y. He , M. Wen , F. M. Wu , D. M. Sun , S. J. Li , L. H. Zhang , Y. M. Li , C. L. Tian , Chem. Commun. 2014, 50, 5837–5839; 10.1039/c4cc00779d 24619065

[4f] J. Li , Y. Li , Q. He , Y. Li , H. Li , L. Liu , Org. Biomol. Chem. 2014, 12, 5435–5441; 24934931 10.1039/c4ob00715h

[4g] S. Tang , Y. Y. Si , Z. P. Wang , K. R. Mei , X. Chen , J. Y. Cheng , J. S. Zheng , L. Liu , Angew. Chem. Int. Ed. 2015, 54, 5713–5717; 10.1002/anie.201500051 25772600

[4h] S. K. Maity , M. Jbara , S. Laps , A. Brik , Angew. Chem. Int. Ed. 2016, 55, 8108–8112; 10.1002/anie.201603169 27126503

[4i] C. Zuo , B. C. Zhang , B. J. Yan , J. S. Zheng , Org. Biomol. Chem. 2019, 17, 727–744. 30566163 10.1039/c8ob02610f

[5a] D. Bang , B. L. Pentelute , S. B. H. Kent , Angew. Chem. Int. Ed. 2006, 45, 3985–3988; 10.1002/anie.200600702 16639756

[5b] G. M. Fang , J. X. Wang , L. Liu , Angew. Chem. Int. Ed. 2012, 51, 10347–10350; 10.1002/anie.201203843 22968928

[5c] T. Durek , V. Y. Torbeev , S. B. H. Kent , Proc. Natl. Acad. Sci. USA 2007, 104, 4846–4851; 17360367 10.1073/pnas.0610630104 PMC1829227

[5d] V. Y. Torbeev , S. B. H. Kent , Angew. Chem. Int. Ed. 2007, 46, 1667–1670; 10.1002/anie.200604087 17397076

[5e] M. Seenaiah , M. Jbara , S. M. Mali , A. Brik , Angew. Chem. Int. Ed. 2015, 54, 12374–12378; 10.1002/anie.201503309 26079184

[6a] L. Velluz , J. Valls , J. Mathieu , Angew. Chem. Int. Ed. Engl. 1967, 6, 778–789;

[6b] J. B. Hendrickson , J. Am. Chem. Soc. 1977, 99, 5439–5450.

[7a] G. M. Fang , Y. M. Li , F. Shen , Y. C. Huang , J. B. Li , Y. Lin , H. K. Cui , L. Liu , Angew. Chem. Int. Ed. 2011, 50, 7645–7649; 10.1002/anie.201100996 21648030

[7b] J. B. Blanco-Canosa , P. E. Dawson , Angew. Chem. Int. Ed. 2008, 47, 6851–6855; 10.1002/anie.200705471 PMC3182823 18651678

[7c] J. X. Wang , G. M. Fang , Y. He , D. L. Qu , M. Yu , Z. Y. Hong , L. Liu , Angew. Chem. Int. Ed. 2015, 54, 2194–2198; 10.1002/anie.201408078 25475965

[7d] J. Mannuthodikayil , S. Singh , A. Biswas , A. Kar , W. Tabassum , P. Vydyam , M. K. Bhattacharyya , K. Mandal , Org Lett. 2019, 21, 9040–9044; 31663760 10.1021/acs.orglett.9b03440

[7e] For a recent report on an alternative to NaNO_2_ treatment for thioester generation from hydrazide, *see* D. T. Flood , J. C. J. Hintzen , M. J. Bird , P. A. Cistrone , J. S. Chen , P. E. Dawson , Angew. Chem. Int. Ed. 2018, 57, 11634–11639; 10.1002/anie.201805191 PMC6126375 29908104

[8] L. A. Carpino , G. Y. Han , J. Org. Chem. 1972, 37, 3404–3409.

[9a] R. Tylercross , V. Schirch , J. Biol. Chem. 1991, 266, 22549–22556; 1939272

[9b] R. C. Stephenson , S. Clarke , J. Biol. Chem. 1989, 264, 6164–6170. 2703484

[10a] K. Harata , M. Muraki , Y. Jigami , J. Mol. Biol. 1993, 233, 524–535; 8105095 10.1006/jmbi.1993.1529

[10b] D. Canet , M. Sunde , A. M. Last , A. Miranker , A. Spencer , C. V. Robinson , C. M. Dobson , Biochemistry 1999, 38, 6419–6427; 10350460 10.1021/bi983037t

[10c] C. Pleyer , J. Flesche , F. Saeed , Clin. Nephrol. Case Stud. 2015, 3, 42–45; 29043133 10.5414/CNCS108538 PMC5437999

[10d] G. W. Kenner , Proc. R. Soc. London Ser. B 1977, 197, 237–253; 19745 10.1098/rspb.1977.0068

[10e] J. J. Sharp , A. B. Robinson , M. D. Kamen , J. Am. Chem. Soc. 1973, 95, 6097–6108; 4733833 10.1021/ja00799a043

[10f] J. Higo , M. Nakasako , J. Comput. Chem. 2002, 23, 1323–1336. 12214315 10.1002/jcc.10100

[11] P. D. Adams , P. V. Afonine , G. Bunkoczi , V. B. Chen , I. W. Davis , N. Echols , J. J. Headd , L. W. Hung , G. J. Kapral , R. W. Grosse-Kunstleve , A. J. McCoy , N. W. Moriarty , R. Oeffner , R. J. Read , D. C. Richardson , J. S. Richardson , T. C. Terwilliger , P. H. Zwart , Acta Crystallogr. Sect. D 2010, 66, 213–221. 20124702 10.1107/S0907444909052925 PMC2815670

