# Supplementary material for: Efficient Chemical Protein Synthesis using Fmoc‐Masked N‐Terminal Cysteine in Peptide Thioester Segments
Source: Angew Chem Int Ed Engl. 2020 May 26;59(35):14796–801. doi: 10.1002/anie.202000491 (PMC7891605; doi:10.1002/anie.202000491)
Supplement: Supplementary file 1 — Supplementary [file ANIE-59-14796-s001.pdf]

Angewandte Chemie
Eine Zeitschrift der Gesellschaft Deutscher Chemiker

Supporting Information

Efficient Chemical Protein Synthesis using Fmoc-Masked N-Terminal Cysteine in Peptide Thioester Segments

*Abhisek Kar[+], Jamsad Mannuthodikayil[+], Sameer Singh, Anamika Biswas, Puneet Dubey, Amit Das, and Kalyaneswar Mandal**

anie_202000491_sm_miscellaneous_information.pdf

1. General Methods ... 3
1.1. Reagents ... 3
1.2. Reverse-phase HPLC and LC-MS analysis ... 3
1.3. A general protocol for machine-assisted Fmoc-SPPS ... 3
2. Model peptides synthesis ... 4
2.1. Synthesis of *Fmoc*-Cys-Leu-Tyr-Arg-Ala-Tyr-$^{\alpha}$*CONHNH$_2$* (1) ... 4
2.2. Synthesis of *Fmoc*-Cys-Trp-Arg-Arg-$^{\alpha}$*CONH$_2$* (I) ... 4
2.3. Synthesis of *Fmoc*-Cys(D)-Trp-Arg-Arg-$^{\alpha}$*CONH$_2$* (II) ... 4
2.4. Synthesis of Ala-Asn-Gly-Trp-Arg-Arg-$^{\alpha}$*CONH$_2$* (III) ... 5
2.5. Synthesis of Ala-Asp-Gly-Trp-Arg-Arg-$^{\alpha}$*CONH$_2$* (IV) ... 5
2.6. Synthesis of model peptide Gly-Cys-Pro-Arg-Ile-Leu-Met-Arg-$^{\alpha}$*COSR'* (3) ... 6
3. Optimization of Fmoc-deprotection condition ... 6
3.1. Effect of pH on Fmoc-deprotection ... 6
3.2. Effect of pH on Fmoc-deprotection in presence of 20% piperidine ... 7
3.3. Effect of piperidine concentration on Fmoc-deprotection at pH 11 ... 8
3.4. Effect of TCEP concentration on Fmoc-deprotection ... 9
4. Compatibility study of peptide sequence in presence of 20% piperidine. ... 9
4.1 Compatibility of Asn-Gly and Asp-Gly sequence ... 9
4.2. Compatibility of N-terminal cysteine residue against racemization ... 11
5. Test ligation of model peptides 1 and 3 in presence of 20% piperidine in ligation buffer ... 11
6. Synthesis of *Pf*-AMA1 polypeptide Cys^{217}-Cys^{302}– $^{\alpha}$*COOH* (11) via One-Pot Ligation ... 12
6.1. Synthesis of *Pf*-AMA1 (3D7) peptide segment Cys^{275}-Cys^{302}- $^{\alpha}$*COOH* (5) ... 12
6.2. Synthesis of *Pf*-AMA1 (3D7 strain) peptide segment *Fmoc*-Cys^{247}-Phe^{274}-$^{\alpha}$*COSR'* (6) ... 12
6.3. Synthesis of *Pf*-AMA1 (3D7) peptide segment *Fmoc*-Cys^{217}-Lys^{246}- $^{\alpha}$*COSR'* (7) ... 13
6.4. One-Pot multi-segment synthesis of *Pf*-AMA1-polypeptide Cys^{217}-Cys^{302}- $^{\alpha}$*COOH* (11) ... 14
7. One-pot total chemical synthesis of human lysozyme ... 14
7.1. Synthesis of lysozyme segment Cys^{95}-Val^{130}- $^{\alpha}$*COOH* (12) ... 14
7.2. Synthesis of lysozyme segment *Fmoc*-Cys^{65}-Ala^{94}-$^{\alpha}$*COSR'* (13) ... 15
7.3. Synthesis of lysozyme segment *Fmoc*-Cys^{30}-Trp^{64}-$^{\alpha}$*COSR'* (14a) ... 16
7.4. Synthesis of lysozyme segment Lys^{1}-Met^{29}-$^{\alpha}$*COSR'* (15) ... 16
7.5. One-pot four-segment ligation of human lysozyme peptide segment 12, 13, 14a and 15 ... 17
8. Convergent synthesis of human lysozyme ... 18
8.1. Synthesis of lysozyme segment Cys^{30}-Trp^{64}- $^{\alpha}$*CONHNH$_2$* (14b) ... 18
8.2. Four segment convergent synthesis of human lysozyme from peptide segment 12, 13, 14b and 15 ... 19
9. Folding and characterization of chemically synthesized human lysozyme ... 21
9.1. Oxidative folding of Lysozyme ... 21
9.2. Crystallization and X-ray structure determination of the synthetic human Lysozyme ... 21
References ... 22

1. General Methods

1.1. Reagents

N,N-Diisopropylethylamine (DIEA), Tris(2-carboxyethyl)phosphine hydrochloride (TCEP), Guanidine hydrochloride (Gu.HCl), Ethyl cyanohydroxyiminoacetate (Oxyma), 4-mercaptophenylacetic acid (MPAA) and all the N$^{\alpha}$-Fmoc protected amino acids were obtained from Chem-Impex International, USA. The side-chain protecting groups used were, Asp(O^{t}Bu), Glu(O^{t}Bu), Asn(Trt), Arg(Pbf), Ser(^{t}Bu), Thr(^{t}Bu), Tyr(^{t}Bu) and *Fmoc*-(Dmb)Gly-OH. *Fmoc*-Cys(Trt)-OH was purchased from Gyros Protein Technologies. *N,N*-Dimethylformamide (DMF, HPLC grade), dichloromethane (DCM, HPLC grade), diethyl ether (AR grade), *N,N′*-diisopropylcarbodiimide (DIC, HPLC grade), and trifluoroacetic acid (TFA, HPLC grade) were purchased from SRL chemicals India. The HPLC grade and LCMS grade acetonitrile (CH_3CN) were purchased from Thermofisher Scientific, India. Piperidine was obtained from AVRA chemicals, India. 2-Chlorotrityl chloride (2-Cl-(Trt)-Cl) resin and *Fmoc*-Rink-Amide resin was purchased from Supra Sciences, India. Sodium 2-mercaptoethanesulfonate (MESNa) and all other common reagents were purchased from Sigma-Aldrich and were of the purest grade available.

1.2. Reverse-phase HPLC and LC-MS analysis

Analytical reverse-phase (RP) HPLC was performed on an Agilent HPLC instrument using an Agilent zorbax SB-C3 (5 µm), 4.6×150 mm reverse-phase silica column at a flow rate of 0.9 mL/min using a linear gradient of 10-54% solvent B in solvent A over 22 min or 10-64% solvent B in solvent A over 27 min at 40 °C (solvent A= 0.1% TFA in H_2O; solvent B = 0.08% TFA in acetonitrile). The UV absorbance of the column eluent was monitored at 214 nm wavelength. The peptide masses were measured over the entire UV absorption peaks corresponding to the compounds being characterized by on-line LC-MS using an Agilent 1290 infinity II/6530 Q-TOF LC/MS instrument. The deconvolution of the charge states of the observed mass was carried out using Agilent MassHunter Qualitative Analysis software (version B.07.00), and the deconvoluted mass of the most abundant isotopologue has been reported with an uncertainty of ± 0.01 Da, unless stated otherwise. Calculated masses were based on average isotope composition or based on the most abundant isotopologue mass determined from the isotopic distribution provided by Agilent MassHunter Qualitative Analysis software.

Preparative reverse phase HPLC (RP-HPLC) of crude peptides was performed with a Waters 1525 preparative HPLC system using Waters C4 (5 µm, 300 Å, 10 × 250 mm) or Agilent ZORBAX-SB C3 (5 µm, 80 Å, 9.4 × 250 mm) columns at 40 °C using an appropriate shallow gradient of increasing concentration of solvent B (0.08% TFA in acetonitrile) in solvent A (0.1% TFA in water) at a flow rate of 5 mL/min. Fractions containing the purified target peptide were identified by ESI-MS. Selected pure fractions were then pooled and lyophilized.

1.3. A general protocol for machine-assisted Fmoc-SPPS

All peptides were synthesized using an automated peptide synthesizer (Tribute-UV/IR from Protein Technologies, USA). Fmoc-SPPS was carried out following reported[1] protocol with minor modifications, using amino acids (AA) (0.25 M), DIC (0.25 M) as a coupling reagent and Oxyma (0.25 M) with DIEA (0.025 M) as additives. Cysteine was coupled for 2 min at room temperature followed by 5 min at 50 °C and Arginine was coupled for 20 min at room temperature followed by 5 min at 50 °C. All other amino acid coupling on the 2-Cl-(Trt)-Cl resin was performed for 6 min at 50 °C under N_2 atmosphere with vortex mixing, and coupling on the *Fmoc*-Rink-Amide resin was performed for 5 min at 65 °C under N_2 atmosphere with vortex mixing. For the synthesis of peptide sequences containing Asp-Gly, which is prone to aspartimide formation during Fmoc-SPPS at elevated temperatures, *Fmoc*-(Dmb)Gly-OH was used instead of *Fmoc*-Gly-OH. For the synthesis of C-terminal hydrazide peptide, hydrazine was coupled on the 2-Cl-(Trt)-Cl resin by adding 10% (vol/vol) hydrazine in DMF and gently agitating for 30 min.[2] The unreacted functional group on 2-Cl-(Trt)-Cl resin was capped using 5% MeOH (vol/vol) in DMF. The coupling of amino acids on NH_2NH-2-Cl-(Trt)-resin was performed in a peptide synthesizer following the coupling protocol mentioned above. Fmoc deprotection after every coupling cycle was carried out by 20% piperidine treatment at 50 °C for 1 min followed

by 3 min at room temperature. After synthesis, the peptides were cleaved from the resin using TFA (85%), Phenol (5%), TIPS (2.5%), Water (5%) and DODT (2.5%) as a cleavage cocktail. After cleavage, the TFA was evaporated under N_2 flow inside a well-ventilated fume hood. The cleaved peptide was precipitated by adding diethyl ether. The resulting precipitate was further washed two times with diethyl ether. Dry crude peptides were either dissolved in 6 M Gu.HCl and loaded directly on to a preparative HPLC column for purification or used for successive reactions without further purification.

2. Model peptide syntheses

2.1. Synthesis of *Fmoc*-Cys-Leu-Tyr-Arg-Ala-Tyr-$^{\alpha}$*CONHNH₂* (1)

The model peptide *Fmoc*-Cys-Leu-Tyr-Arg-Ala-Tyr-$^{\alpha}CONHNH_2$ (**1**) was synthesized on NH_2NH-2-Cl-(Trt)-resin (substitution = 0.67 mmol/g) by stepwise Fmoc chemistry SPPS on a 0.2 mmol scale in an automated peptide synthesizer (see **Section 1.3** for the peptide synthesis protocol). The Fmoc group of the N-terminal cysteine residue was left on during the global deprotection of peptide from the resin. Purification using preparative HPLC gave 78 mg (76 µmol, 38% yield) of the pure peptide **1** (**Figure S1a**). Observed mass (ESI-MS): 1023.47 Da (deconvoluted monoisotopic peak); calculated mass: 1023.47 Da (monoisotopic).

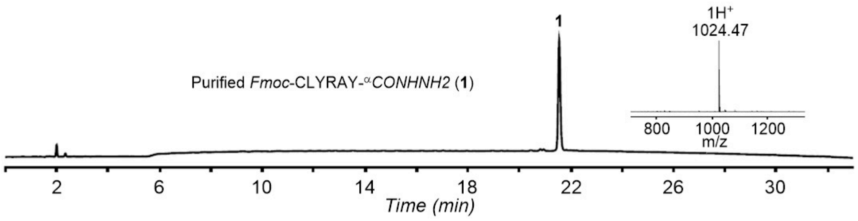

Figure S1a. Analytical RP-HPLC profile (λ = 214 nm) together with ESI-MS data (inset) of purified model peptide *Fmoc*-Cys-Leu-Tyr-Arg-Ala-Tyr-$^{\alpha}CONHNH_2$ (**1**) synthesized using NH_2NH-2-Cl-(Trt)-resin. Linear gradient 10-70% of B in buffer A (buffer A = 0.1% TFA in water; buffer B = 0.08% TFA in acetonitrile) at 40 °C over 30 min including 4 min equilibration time using Agilent Zorbax SB-C3, 5 µm 4.6 × 150 mm, LC column with 0.9 mL/min flow rate was used for the chromatographic separation. Purification was performed using a linear gradient 25%-45% buffer B in buffer A over 40 min with a flow rate of 5 mL/min at 40 °C using a C4, 10 × 250 mm, preparative HPLC column (Phenomenex proteo, 300 Å, 10 µm).

2.2. Synthesis of *Fmoc*-Cys-Trp-Arg-Arg-$^{\alpha}$*CONH₂* (I)

The model peptide *Fmoc*-Cys-Trp-Arg-Arg-$^{\alpha}CONH_2$ (**I**) was synthesized on Rink-Amide resin (substitution = 0.58 mmol/g) by stepwise Fmoc chemistry SPPS on a 0.1 mmol scale in an automated peptide synthesizer (see **Section 1.3** for the peptide synthesis protocol). The Fmoc group of the N-terminal cysteine residue was left on during the global deprotection of peptide from the resin. Observed mass (ESI-MS): 840.40 Da (deconvoluted monoisotopic peak); calculated mass: 840.39 Da (monoisotopic).

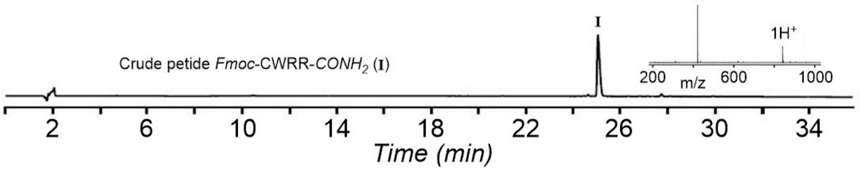

Figure S1b. Analytical RP-HPLC profile (λ = 214 nm) together with ESI-MS data (inset) of the crude model peptide *Fmoc*-Cys-Trp-Arg-Arg-$^{\alpha}CONH_2$ (**I**). Linear gradient 1%-61% B in buffer A (buffer A = 0.1% TFA in water; buffer B = 0.08% TFA in acetonitrile) at 40 °C over 30 min including 4 min equilibration time using Agilent Zorbax SB-C3, 5 µm, 4.6 × 150 mm, LC column with 0.9 mL/min flow rate was used for the chromatographic separation.

2.3. Synthesis of *Fmoc*-Cys(D)-Trp-Arg-Arg-$^{\alpha}$*CONH₂* (II)

The model peptide *Fmoc*-Cys(D)-Trp-Arg-Arg-$^{\alpha}CONH_2$ (**II**) was synthesized on Rink-Amide resin (substitution = 0.58 mmol/g) by stepwise Fmoc chemistry SPPS on a 0.1 mmol scale in an automated peptide synthesizer (see **Section 1.3** for the peptide synthesis

protocol). The Fmoc group of the N-terminal cysteine residue was left on during the global deprotection of peptide from the resin. Observed mass (ESI-MS): 840.40 Da (deconvoluted monoisotopic peak); calculated mass: 840.39 Da (monoisotopic).

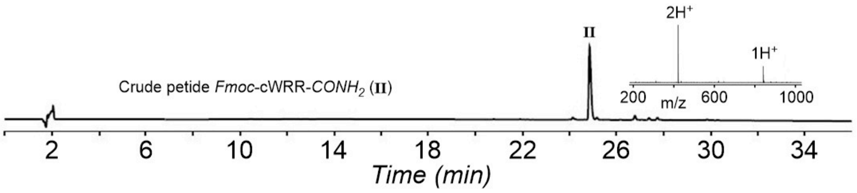

Figure S1c. Analytical RP-HPLC profile (λ = 214 nm) together with ESI-MS data (inset) of the crude model peptide *Fmoc*-Cys(D)-Trp-Arg-Arg-$^{\alpha}$*CONH*$_2$ (**II**). Linear gradient 1%-61% B in buffer A (buffer A = 0.1% TFA in water; buffer B = 0.08% TFA in acetonitrile) at 40 °C over 30 min including 4 min equilibration time using Agilent Zorbax SB-C3, 5 µm, 4.6 × 150 mm, LC column with 0.9 mL/min flow rate was used for the chromatographic separation.

2.4. Synthesis of Ala-Asn-Gly-Trp-Arg-Arg-$^{\alpha}$*CONH*$_2$ (III)

The model peptide Ala-Asn-Gly-Trp-Arg-Arg-$^{\alpha}$*CONH*$_2$ (**III**) was synthesized on Rink-Amide resin (substitution = 0.58 mmol/g) by stepwise Fmoc chemistry SPPS on a 0.1 mmol scale in an automated peptide synthesizer (see **Section 1.3** for the peptide synthesis protocol). Purification using preparative HPLC gave the pure peptide **III** (**Figure S1d**). Observed mass (ESI-MS): 757.41 Da (deconvoluted monoisotopic peak); calculated mass: 757.41 Da (monoisotopic).

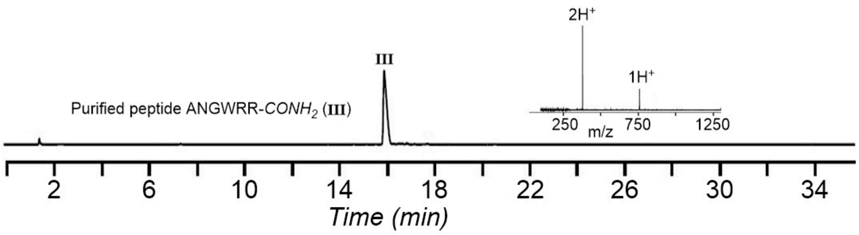

Figure S1d. Analytical RP-HPLC profile (λ = 214 nm) together with ESI-MS data (inset) of the purified model peptide Ala-Asn-Gly-Trp-Arg-Arg-$^{\alpha}$*CONH*$_2$ (**III**). Linear gradient 01-31% of B in buffer A (buffer A = 0.1% TFA in water; buffer B = 0.08% TFA in acetonitrile) at 40 °C over 30 min including 4 min equilibration time using Agilent Zorbax SB-C8, 3.5 µm, 4.6 × 150 mm, LC column with 0.9 mL/min flow rate was used for the chromatographic separation. Purification was performed using a linear gradient 01%-41% buffer B in buffer A over 80 min with a flow rate of 5 mL/min at 40 °C using a C4, 10 × 250 mm, preparative HPLC column (Phenomenex proteo, 300 Å, 10 µm).

2.5. Synthesis of Ala-Asp-Gly-Trp-Arg-Arg-$^{\alpha}$*CONH*$_2$ (IV)

The model peptide Ala-Asp-Gly-Trp-Arg-Arg-$^{\alpha}$*CONH*$_2$ (**IV**) was synthesized on Rink-Amide resin (substitution = 0.58 mmol/g) by stepwise Fmoc chemistry SPPS on a 0.1 mmol scale in an automated peptide synthesizer (see **Section 1.3** for the peptide synthesis protocol). Purification using preparative HPLC gave the pure peptide **IV** (**Figure S1e**). Observed mass (ESI-MS): 758.40 Da (deconvoluted monoisotopic peak); calculated mass: 758.39 Da (monoisotopic).

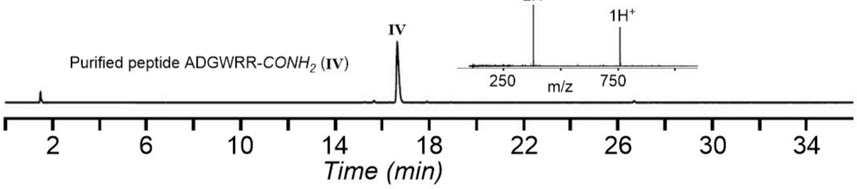

Figure S1e. Analytical RP-HPLC profile (λ = 214 nm) together with ESI-MS data (inset) of the purified model peptide Ala-Asp-Gly-Trp-Arg-Arg-$^{\alpha}$*CONH*$_2$ (**IV**). Linear gradient 01-31% of B in buffer A (buffer A = 0.1% TFA in water; buffer B = 0.08% TFA in acetonitrile) at 40 °C over 30 min including 4 min equilibration time using Agilent Zorbax SB-C8, 3.5 µm, 4.6 × 150 mm, LC column with 0.9 mL/min flow rate was used for the chromatographic separation. Purification was performed using a linear gradient 01%-41% buffer B in buffer A over 80 min with a flow rate of 5 mL/min at 40 °C using a C4, 10 × 250 mm, preparative HPLC column (Phenomenex proteo, 300 Å, 10 µm).

2.6. Synthesis of model peptide Gly-Cys-Pro-Arg-Ile-Leu-Met-Arg-$^{\alpha}$*COSR'* (3)

The corresponding hydrazide peptide Gly-Cys-Pro-Arg-Ile-Leu-Met-Arg-$^{\alpha}$*CONHNH$_2$* (**3'**) was first synthesized using NH_2NH-2-Cl-(Trt)-resin (substitution = 0.62 mmol/g) by stepwise Fmoc chemistry SPPS (0.2 mmol scales) in an automated peptide synthesizer (see **Section-1.3** for the peptide synthesis protocol). After global deprotection using TFA cocktail, the peptide hydrazide was precipitated using diethyl ether. The mass of the crude peptide **3'** was confirmed by LC-MS (**Figure S2-a)**; Observed mass (ESI-MS): 958.54 Da (deconvoluted most abundant isotopologue, monoisotopic); calculated mass: 958.53 Da (monoisotopic). Crude peptide **3' (**~170 mg, ~0.2 mmol) was then dissolved in 8 mL of aqueous phosphate buffer (0.2 M) containing 6 M Gu.HCl at pH 3.0 and incubated at -16.5 °C (using Julabo). After 15 min, 800 µL of aqueous $NaNO_2$ (0.5 M) was added to the solution of peptide **3'** and gently agitated for 15 min at -16.5 °C. Afterward, 8 mL of 0.2 M MESNa containing 0.2 M aqueous phosphate buffer and 6 M Gu.HCl at pH 6.3 was mixed into the oxidized solution of peptide **3'** and the temperature was raised to room temperature. The pH was adjusted to 6.5 and the MESNa exchange was complete within 20 min as monitored by LCMS (**Figure S2-b)**. Finally, 241 mg of TCEP (~50 mM) was added as solid powder and the pH of the resulting reaction mixture was adjusted to 3.5 and agitated for 30 min to reduce the oxidized cysteines in the peptide before purifications. (Note: Incubation at pH higher than 4.5 produced thiolactone (-32 Da from the peptide hydrazide mass); hence, the reaction mixture should be agitated at pH <4 for a longer time in case of incomplete disulfide reduction). Purification using preparative HPLC afforded 56 mg (58.42 µmol, 29.2% yield) of the desired MESNa exchanged peptide **3** (**Figure S2-c**). Observed mass (ESI-MS): 1068.48 Da (deconvoluted most abundant isotopologue, monoisotopic); calculated mass: 1068.48 Da (monoisotopic).

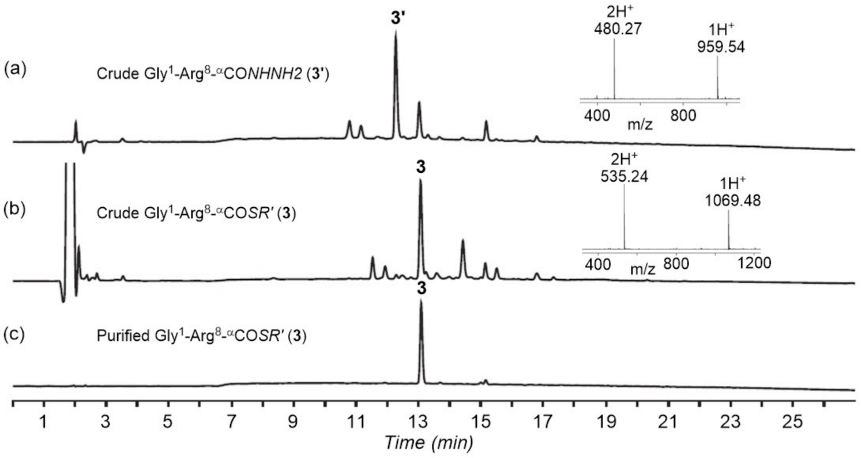

Figure S2. Analytical RP-HPLC profile (λ = 214 nm) together with ESI-MS data (inset) for the synthesis of peptide Gly-Cys-Pro-Arg-Ile-Leu-Met-Arg-$^{\alpha}$*COSR'* (**3**). (a) Chromatogram of the crude peptide Gly-Cys-Pro-Arg-Ile-Leu-Met-Arg-$^{\alpha}$*CONHNH$_2$* (**3'**). (b) Chromatogram of the MESNa exchanged product Gly-Cys-Pro-Arg-Ile-Leu-Met-Arg-$^{\alpha}$*COSR'* (**3**) from the crude peptide **3'**. (c) Chromatogram for the purified peptide Gly-Cys-Pro-Arg-Ile-Leu-Met-Arg-$^{\alpha}$*COSR'* (**3**). R' = *–$CH_2CH_2SO_3Na$* Linear gradient 10%-54% of B over 22 min including 4 min equilibration using Agilent Zorbax SB-C3, 5 µm, 4.6 × 150 mm LC column with 0.9 mL/min flow rate was used for all the chromatographic separation. Purification was performed using a linear gradient 5%-25% buffer B in buffer A over 60 min with a flow rate of 5 mL/min at 40 °C (buffer A = 0.1% TFA in water; buffer B = 0.08% TFA in acetonitrile) using a C4, 10 × 250 mm column (Phenomenex proteo, 300 Å, 10 µm).

3. Optimization of Fmoc-deprotection condition

3.1. Effect of pH on Fmoc-deprotection

To check the effect of pH on Fmoc-deprotection, model peptide *Fmoc*-Cys-Leu-Tyr-Arg-Ala-Tyr-$^{\alpha}$*CONHNH$_2$* (**1**) was dissolved (1 mg/mL) in three different buffers of pH 9.0, 10.0 and 11.0, separately. The buffers consisted of aqueous sodium phosphate (0.2 M) containing 6 M Gu.HCl and 20 mM TCEP. At pH 9.0 and 10.0, the deprotection kinetics of the Fmoc-group was found to be extremely slow. However, the complete Fmoc-removal was observed after 9 h at pH 11.0 (**Figure S3**). The identity of the Fmoc deprotected product (**2**) was confirmed by ESI-MS (Observed mass: 801.40 Da (deconvoluted most abundant isotopologue, monoisotopic); calculated mass: 801.39 Da (monoisotopic)). The anticipated piperidine adduct with dibenzo fulvene (#) (Observed mass: 263.17 Da (monoisotopic);

calculated mass: 263.16 Da (monoisotopic)) and a new side product, the TCEP adduct with dibenzo fulvene (*) (Observed mass (ESI-MS): 428.14 Da (monoisotopic); calculated mass: 428.14 Da (monoisotopic)), were observed during the deprotection.

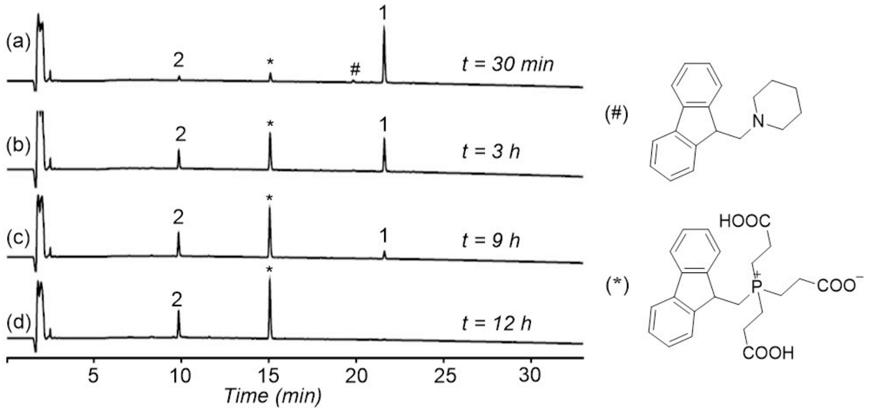

Figure S3. Analytical RP-HPLC profile (λ = 214 nm) monitoring together with ESI-MS data (inset) of the Fmoc deprotection of the model peptide *Fmoc*-Cys-Leu-Tyr-Arg-Ala-Tyr-$^{\alpha}$*CONHNH*$_2$ **(1)** in 200 mM PB, 6 M Gu.HCl, 20 mm TCEP at pH 11.0. Chromatogram after 30 min (a); after 3 h (b); after 9 hr (c); after 12 hr (d). Linear gradient 10%-64% of B over 27 min including 4 min equilibration time using Agilent Zorbax SB-C3, 5 µm, 4.6 × 150 mm LC column with 0.9 mL/min flow rate was used for all the chromatographic separations. '#' indicates piperidine adduct with dibenzofulvene and '*' indicates the TCEP adduct with dibenzofulvene.

3.2. Effect of pH on Fmoc-deprotection in presence of 20% piperidine

To check the effect of pH on Fmoc-deprotection in presence of 20% piperidine, the model peptide *Fmoc*-Cys-Leu-Tyr-Arg-Ala-Tyr-$^{\alpha}$*CONHNH*$_2$ **(1)** (1.5 mg, 1.46 µmol) was dissolved in 1.2 mL of aqueous phosphate buffer (0.2 M) containing 6 M Gu.HCl and 20 mM TCEP (pH 7) and divided into three equal portions. To each reaction mixture were added piperidine and HCl (conc.) in such a way that the final concentration of piperidine remained 20% and the pH the three reaction mixtures was 9.0, 10.0 and 11.0, respectively. The progress of the Fmoc group deprotection was monitored by HPLC from a small aliquot of the reaction mixture taken at different time intervals. The percentage of conversion was calculated from the area under the curve obtained from the 214 nm UV absorption spectra in RP-HPLC (**Figure S4a**). Two byproducts of the cleaved Fmoc-group, the piperidine adduct with dibenzofulvene (#) (Observed mass (ESI-MS): 263.17 Da (monoisotopic); calculated mass: 263.16 Da) and the TCEP adduct with dibenzofulvene (*) (Observed mass (ESI-MS): 428.14 Da (monoisotopic); calculated mass: 428.14 Da) were observed during the deprotection. Notably, the piperidine adduct with dibenzofulvene (#) was completely converted to the TCEP adduct with dibenzofulvene **(*),** within two hours.

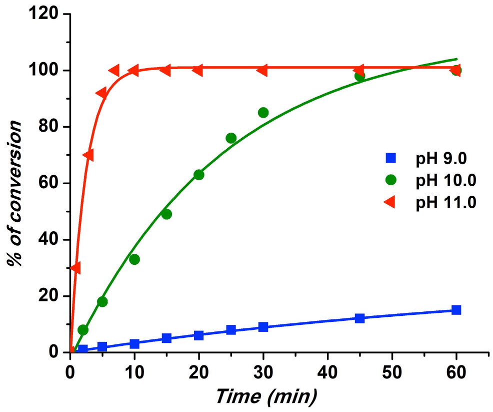

Figure S4a. Fmoc deprotection kinetics in presence of 20% piperidine in aqueous ligation buffer 200 mM PB, 6 M Gu. HCl, 20 mM TCEP at pH 9 (■), 10 (●) and 11 (▲). Solid lines represent the fitted plot over experimental data points.

To test the stability of the Fmoc group in native chemical ligation condition in presence of 20% piperidine, the same model peptide *Fmoc*-Cys-Leu-Tyr-Arg-Ala-Tyr- $^{\alpha}$*CONHNH*$_2$ **(1)** (1 mg, 0.97 µmol) was dissolved in 1 mL of aqueous phosphate buffer (0.2 M sodium phosphate, 6 M Gu.HCl and 20 mM TCEP, pH 7.0) containing 20% piperidine. The progress of the reaction was monitored by analytical HPLC (**Figure S4b**) from a small aliquot of the reaction mixture taken at different time intervals. The HPLC chromatogram showed no

deprotection of the Fmoc group from the N-terminal cysteine even after 2 days incubation of the peptide in ligation buffer at pH 7 in presence of 20% piperidine.

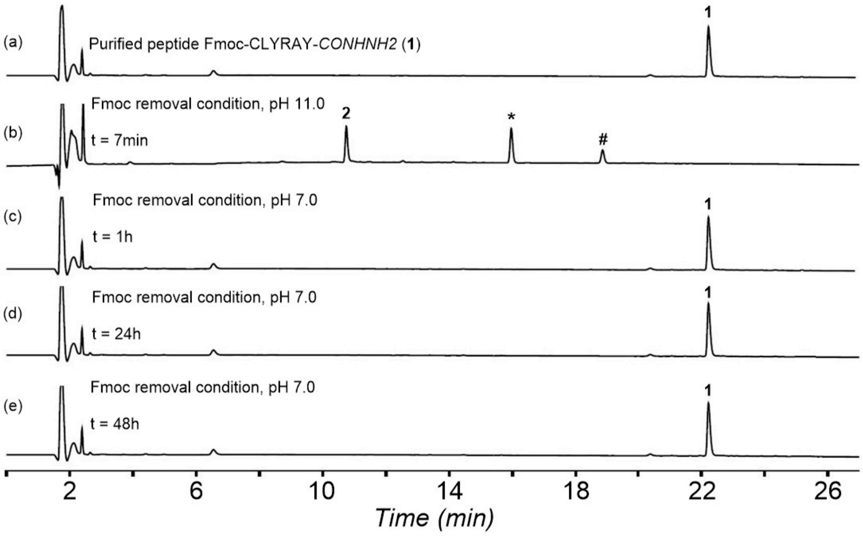

Figure S4b. Analytical RP-HPLC profile (λ = 214 nm) to check the stability of Fmoc group of the model peptide *Fmoc*-Cys-Leu-Tyr-Arg-Ala-Tyr-$^{\alpha}$*CONHNH*$_2$ **(1)** in 200 mM PB, 6 M Gu.HCl, 20 mm TCEP at pH 7.0. (a) Chromatogram of purified model peptide **1**; (b) positive control: the model peptide **1** in ligation buffer in presence of 20% piperidine at pH 11.0 after 7 min. The Fmoc deprotected peptide **2** eluted at 10.90 min. The model peptide in ligation buffer in presence of 20% piperidine after (c) 1h at pH 7.0; (d) after 24h at pH 7.0; (e) after 48h at pH 7.0. Linear gradient 10%-54% of B in buffer A (buffer A = 0.1% TFA in water; buffer B = 0.08% TFA in acetonitrile) at 40 °C over 22 min including 4 min equilibration time using Agilent Zorbax SB-C3, 5 µm, 4.6 × 150 mm LC column with 0.9 mL/min flow rate was used for all the chromatographic separations.

3.3. Effect of piperidine concentration on Fmoc-deprotection at pH 11

To check the effect of piperidine concentration on Fmoc-deprotection at pH 11, model peptide *Fmoc*-Cys-Leu-Tyr-Arg-Ala-Tyr-$^{\alpha}$*CONHNH*$_2$ **(1)** was dissolved in aqueous phosphate buffer (0.2 M) containing 6 M Gu.HCl and 20 mM TCEP. The final peptide concentration was maintained at 1 mg/mL. The pH was adjusted to 7, and the reaction mixture was divided into three equal portions. Piperidine and HCl (conc.) were added to each portion of the reaction mixture to attain pH 11.0, and the final concentration of piperidine in the respective portions to 5%, 10%, and 20%. The progress of the deprotection at different time points was monitored by RP-HPLC and the percentage of conversion was calculated from the area under the curve obtained from the 214 nm UV absorption spectra in RP-HPLC (**Figure S5**).

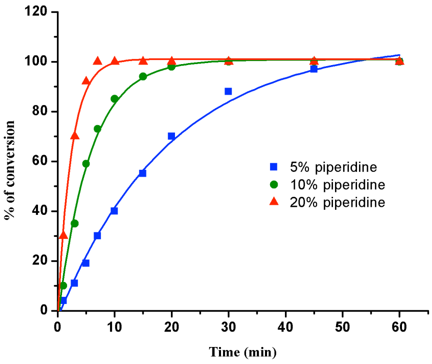

Figure S5. Fmoc deprotection kinetics in presence of 5% (■), 10% (●) and 20% (▲) piperidine in aqueous ligation buffer (200 mM PB, 6 M Gu. HCl, 20 mM TCEP*)* at pH 11. Solid lines represent the fitted plot over experimental data points.

3.4. Effect of TCEP concentration on Fmoc-deprotection

Since we observed dibenzofulvene-TCEP adduct along with the dibenzofulvene-piperidine adduct during the Fmoc deprotection step, we decided to investigate the effect of TCEP concentration on Fmoc-deprotection. As the reaction kinetics was very fast at pH 11 in the presence of 20% piperidine, the influence of TCEP was studied at pH 10. TCEP concentrations of 20 mM, 50 mM, and 100 mM were examined. The model peptide *Fmoc*-Cys-Leu-Tyr-Arg-Ala-Tyr-$^{\alpha}$*CONHNH*$_2$ **(1)** was dissolved in aqueous phosphate buffer (0.2 M) containing 6 M Gu.HCl. The peptide solution (1 mg/mL) was then divided into three equal portions having 20 mM, 50 mM, and 100 mM TCEP, respectively. Piperidine and HCl (conc.) were added to each portion to attain the final concentration of 20% piperidine maintaining pH 10.0 in each portion. We found that the variant concentration of TCEP did not have any effect on Fmoc-deprotection kinetics (**Figure S6**). However, the rate of formation of TCEP adduct with dibenzofulvene was increased with increasing TCEP concentration.

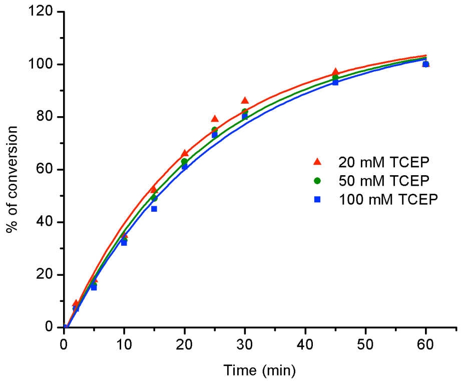

Figure S6. Fmoc deprotection kinetics in aqueous ligation buffer (200 mM PB, 6 M Gu.HCl) in presence of 20% piperidine at pH 10 with 20 mM (▲) 50 mM (●) and 100 mM (■) TCEP concentration.

4. Compatibility study of peptide sequence in presence of 20% piperidine.

4.1 Compatibility of Asn-Gly and Asp-Gly sequence

The peptide sequences containing Asn-Gly or Asp-Gly are known to undergo aspartimide formation at high pH, which upon hydrolysis form deamidated and iso-aspartate peptides. To test the stability of such peptide sequence in presence of 20% piperidine at pH 11 in aqueous buffer (200 mM PB, 6 M Gu.HCl, 20 mM TCEP), we synthesized two model peptides, Ala-Asn-Gly-Trp-Arg-Arg-$^{\alpha}$*CONH*$_2$ (**III**) and Ala-Asp-Gly-Trp-Arg-Arg-$^{\alpha}$*CONH*$_2$ (**IV**) having Asn-Gly and Asp-Gly in their sequence, respectively (see ***Section 2.0*** for model peptide synthesis). Peptide **III** and **IV** were dissolved separately in pre-made buffer (200 mM PB, 6 M Gu.HCl, 20 mM TCEP, pH 11.0) containing 20% piperidine. The progress of the reaction was monitored by LC-MS (**Figure S7a**) from a small aliquot of the reaction mixture taken at different time intervals. LC-MS analysis showed that even after 14 minutes (considering two Fmoc removal steps for four segment one-pot ligation) very minor amount, <4% of the iso-aspartate (**IIIa;** Observed mass (ESI-MS): 758.42 Da (deconvoluted monoisotopic peak); calculated mass: 758.40 Da (monoisotopic)) and <2% of the deamidated peptides (IIIb/**IV;** Observed mass: 758.42 Da (deconvoluted monoisotopic peak), calculated mass: 758.40Da (monoisotopic)), from peptide **III** was observed (**Figure S7a**). There was no detectable amount of iso-aspartate peptide formation from the peptide **IV** was observed even after 60 min incubation at pH 11.0 (**Figure S7b**). The identity of the iso-aspartate (**IIIa**) and the aspartate peptide (**IIIb**) side products originated from the peptide **III** was confirmed by the retention time analysis of the co-elution of both the reaction mixtures after 60 min, as one of the side products from the peptide **III** (deamidated peptide **IIIb**) is identical to peptide **IV** (**Figure S7c**). This co-elution experiment also clearly suggests that there is no significant formation of iso-aspartate from the peptide **IV** as the iso-aspartate peptide (**IIIa**) has a distinct retention time shift (0.2 min) from peptide **IV** in HPLC chromatogram (**Figure S7c**).

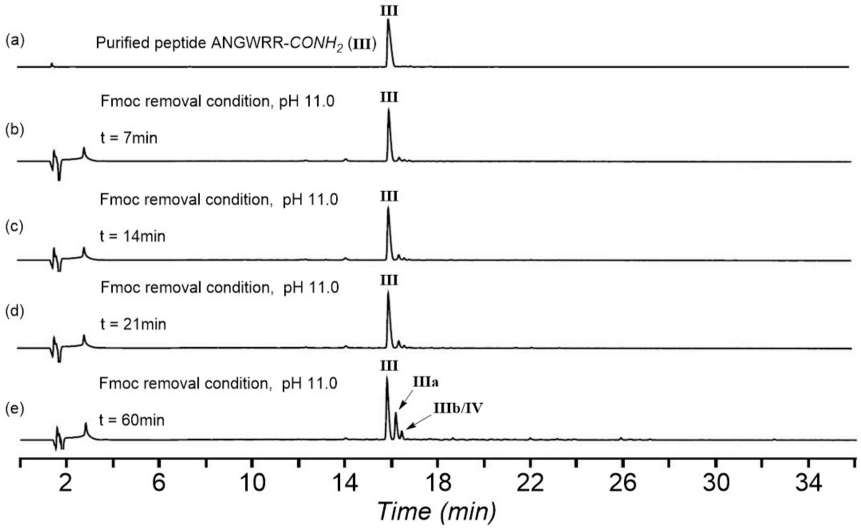

Figure S7a. Analytical HPLC monitoring (λ= 214 nm) of the peptide Ala-Asn-Gly-Trp-Arg-Arg-$^{\alpha}CONH_2$ (**III**) in the buffer containing 200 mM PB, 6 M Gu.HCl, 20 mM TCEP and 20% piperidine at pH 11.0. (a) Purified starting peptide **III**. Peptide **III** after 7 min (b), after 14 min (c), after 21 min (d) and after 60 min (e) incubation in buffer (pH 11.0) containing 20% piperidine. Linear gradient 1-31% of B in buffer A (buffer A = 0.1% TFA in water; buffer B = 0.08% TFA in acetonitrile) at 40 °C over 30 min including 4 min equilibration time using Agilent Zorbax SB-C8, 3.5 µm, 4.6 × 150 mm, LC column with 0.9 mL/min flow rate was used for the chromatographic separations.

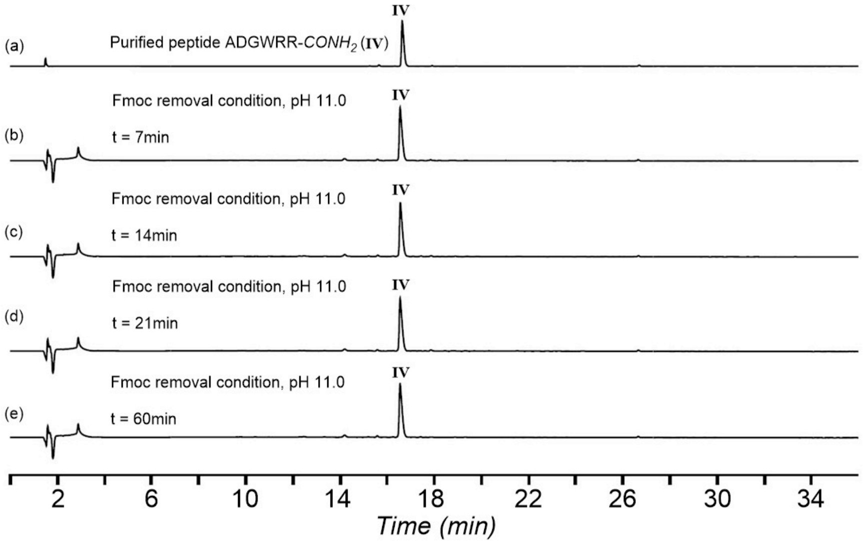

Figure S7b. Analytical HPLC monitoring (λ= 214 nm) of the peptide Ala-Asp-Gly-Trp-Arg-Arg-$^{\alpha}CONH_2$ (**IV**) in the buffer containing 200 mM PB, 6 M Gu.HCl, 20 mM TCEP and 20% piperidine at pH 11.0. (a) Purified peptide **IV**. Peptide **IV** After 7 min (b), after 14 min (c), after 21 min (d) and after 60 min (e) incubation in buffer (pH 11.0) containing 20% piperidine. Linear gradient 1-31% of B in buffer A (buffer A = 0.1% TFA in water; buffer B = 0.08% TFA in acetonitrile) at 40 °C over 30 min including 4 min equilibration time using Agilent Zorbax SB-C8, 3.5 µm 4.6 × 150 mm, LC column with 0.9 mL/min flow rate was used for the chromatographic separation.

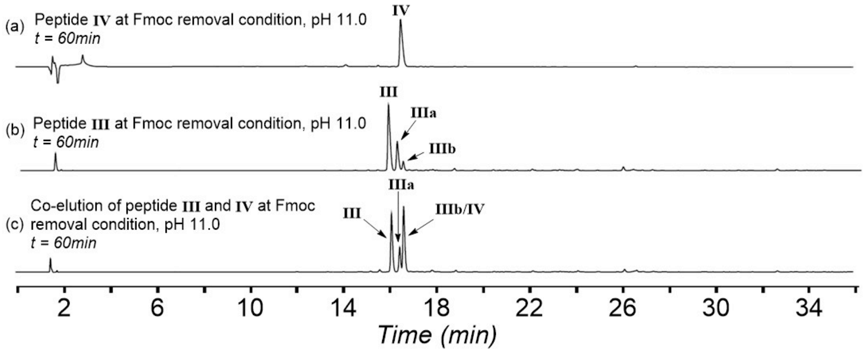

Figure S7c. Co-elution experiment. Analytical HPLC profile (λ= 214 nm) for the co-elution of peptide Ala-Asn-Gly-Trp-Arg-Arg-$^{\alpha}CONH_2$ (**III**) and Ala-Asp-Gly-Trp-Arg-Arg-$^{\alpha}CONH_2$ (**IV**) after keeping peptides in the buffer (200 mM PB, 6 M Gu.HCl, 20 mM TCEP, pH 11.0) containing 20%

piperidine for 60 minutes. Peptide **IV** After 60 min (a), peptide **III** after 60 min (b) and peptide **III** and **IV** together after 60 min (c) of incubation in buffer (pH 11.0) with 20% piperidine. Linear gradient 01-31% of B in buffer A (buffer A = 0.1% TFA in water; buffer B = 0.08% TFA in acetonitrile) at 40 °C over 30 min including 4 min equilibration time using Agilent Zorbax SB-C8, 3.5 µm 4.6 × 150 mm, LC column with 0.9 mL/min flow rate was used for the chromatographic separation.

4.2. Compatibility of N-terminal cysteine residue against epimerization

Base catalyzed epimerization is likely to happen when carboxylic acid group in any amino acid is in activated form. Once the peptide bond is formed, epimerization is less likely to be influenced by any base, such as piperidine used in our Fmoc deprotection condition. In order to confirm the compatibility of N-terminal cysteine residue against racemization during Fmoc removal condition, we performed the Fmoc deprotection of two model peptides, *Fmoc*-Cys-Trp-Arg-Arg-$^{\alpha}$*CONH*$_2$ (**I**) and *Fmoc*-Cys(D)-Trp-Arg-Arg-$^{\alpha}$*CONH*$_2$ (**II**) strategically designed to get significant separation in retention time in the HPLC chromatogram. At first, the Fmoc deprotection of the mixture of peptide **I** and **II** was done in aqueous buffer (200 mM PB, 6 M Gu.HCl, 20 mM TCEP, pH 11.0) containing 20% piperidine. The Fmoc deprotected products from peptide **I** and **II** [**Ia;** Observed mass (ESI-MS): 618.33 Da (deconvoluted monoisotopic peak); calculated mass: 618.32 Da (monoisotopic) and **IIa;** Observed mass (ESI-MS): 618.33 Da (deconvoluted monoisotopic peak); calculated mass: 618.32 Da (monoisotopic)] eluted as two distinct peaks in HPLC chromatogram (**Figure S8**). Next, we performed the same experiment independently with peptide **I** in presence of 20% piperidine (pH 11.0) and monitored the progress of the reaction by LC-MS (**Figure S8**) from a small aliquot of the reaction mixture taken at different time intervals. No epimerization of **Ia** was observed even up to 30 minutes incubation at pH 11.0 in presence of 20% piperidine indicating high compatibility of the N-terminal cysteine residue during Fmoc removal condition used in our experiments.

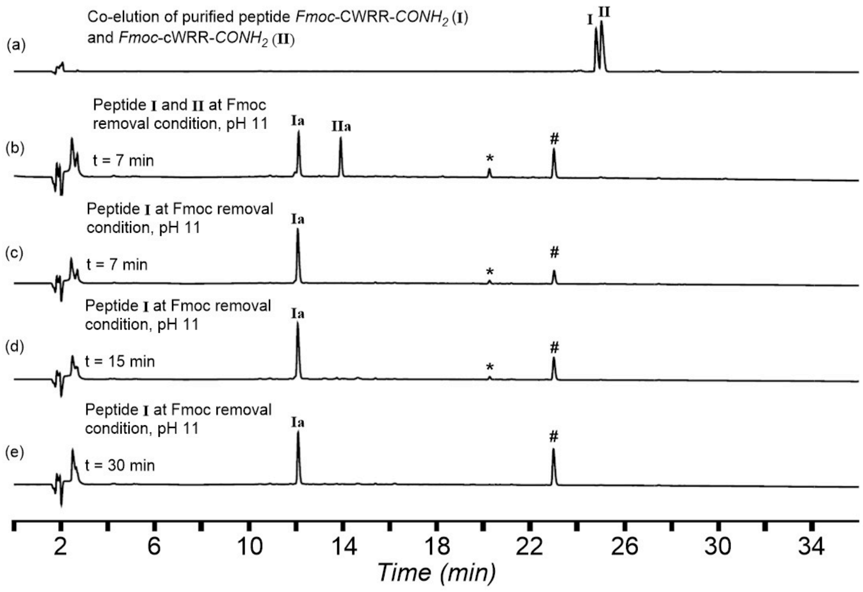

Figure S8. Analytical HPLC monitoring (λ= 214 nm) to test the compatibility of N-terminal cysteine residue in the buffer containing 200 mM PB, 6 M Gu.HCl, 20 mM TCEP and 20% piperidine at pH 11.0. (a) Chromatogram of a mixture of peptide *Fmoc*-Cys-Trp-Arg-Arg-$^{\alpha}$*CONH*$_2$ (**I**) and *Fmoc*-Cys(D)-Trp-Arg-Arg-$^{\alpha}$*CONH*$_2$ (**II**). (b) Chromatogram of the mixture of peptide (**I**) and (**II**) in buffer (pH 11.0) in presence of 20% piperidine after 7 min. **Ia** and **IIa** are the Fmoc deprotected peptides from peptide **I** and **II,** respectively. Chromatogram for the peptide *Fmoc*-Cys-Trp-Arg-Arg-$^{\alpha}$*CONH*$_2$ (**I**) in buffer (pH 11.0) in presence of 20% piperidine after 7 min (c), after 15 min (d) and after 30 min (e) of incubation in buffer (pH 11.0) with 20% piperidine. Linear gradient 1-61% of B in buffer A (buffer A = 0.1% TFA in water; buffer B = 0.08% TFA in acetonitrile) at 40 °C over 30 min including 4 min equilibration time using Agilent Zorbax SB-C3, 5 µm 4.6 × 150 mm, LC column with 0.9 mL/min flow rate was used for the chromatographic separation.

5. Test ligation of model peptides 1 and 3 in presence of 20% piperidine in ligation buffer

To test the feasibility of the native chemical ligation reaction in presence of 20% piperidine, we ligated a model peptide *Fmoc*-Cys-Leu-Tyr-Arg-Ala-Tyr-$^{\alpha}$*CONHNH*$_2$ (**1**), after removing the Fmoc group using piperidine, with Gly-Cys-Pro-Arg-Ile-Leu-Met-Arg-$^{\alpha}$*COSR* (**3**). To a solution of peptide **1** (2 mg, 1.95 µmol) dissolved in 2 mL of ligation buffer (200 mM PB, 6 M Gu.HCl) was added piperidine and the pH of the reaction mixture was adjusted to 11.0 maintaining the final piperidine concentration to 20% (v/v). After 7 minutes (including 2

minutes time required for pH adjustment) the pH was reduced to 7 by adding concentrated HCl followed by TCEP (11.5 mg, 20 mM) as solid powder, Finally, to the reaction mixture, 20 mM MPAA followed by peptide **3** (2.08 mg, 1.95 µmol) were added, and the pH was adjusted to 6.8. The progress of the ligation reaction was monitored by reverse-phase HPLC and the ligation was essentially completed within 10 h resulting in a clean conversion to the ligated product **4** (**Figure 2, main text**). The identity of the desired ligated product **4** was confirmed by LC-MS analysis (Observed mass (ESI-MS): 1727.90 Da (deconvoluted most abundant isotopologue, monoisotopic); calculated mass: 1727.89 Da (monoisotopic)). *[Notes: 1) Careful adjustment of the pH up to 11 does not lead to any precipitate formation. However, we have noticed that overshooting the pH to >11 during pH adjustment leads precipitation, which gets dissolved in lower pH in the next step without hampering the reaction outcome. 2) The pH adjustments were performed at room temperature by portion-wise addition of piperidine (for Fmoc deprotection step), or portion-wise addition of HCL/NaOH (during successive one-pot ligation steps), with vigorous vortexing of the reaction mixture using a vortex mixer to dissipate the generated heat. These exothermic pH adjustments can also be performed at 0°C to prevent local heat release.]*

6. Synthesis of *Pf*-AMA1 polypeptide Cys^{217}-Cys^{302}–$^{\alpha}$*COOH* (11) via One-Pot Ligation

6.1. Synthesis of *Pf*-AMA1 (3D7) peptide segment Cys^{275}-Cys^{302}- $^{\alpha}$*COOH* (5)

The peptide Cys-Phe-Arg-Pro-Ala-Lys-Asp-Ile-Ser-Phe-Gln-Asn-Tyr-Thr-Tyr-Leu-Ser-Lys-Asn-Val-Val-Asp-Asn-Trp-Glu-Lys-Val-Cys-$^{\alpha}$*COOH* (**5**) was synthesized using NH_2NH-2-Cl-(Trt)-resin (scale = 0.3 mmol; substitution = 0.645 mmol/g) by stepwise Fmoc chemistry SPPS in an automated peptide synthesizer at 50 °C (see **Section-1.3** for the peptide synthesis protocol). After global deprotection using the TFA cocktail, the crude peptide **5** was precipitated using diethyl ether. Purification of the crude peptide by preparative HPLC gave 160.7 mg (47.7 µmol, 16%) of the pure peptide segment Cys^{275}-Cys^{302}-$^{\alpha}$*COOH* (**5**). Observed mass (**ESI-MS**): 3368.62 Da (deconvoluted most abundant isotopologue); calculated mass: 3368.62 Da (most abundant isotopologue) (**Figure S9**).

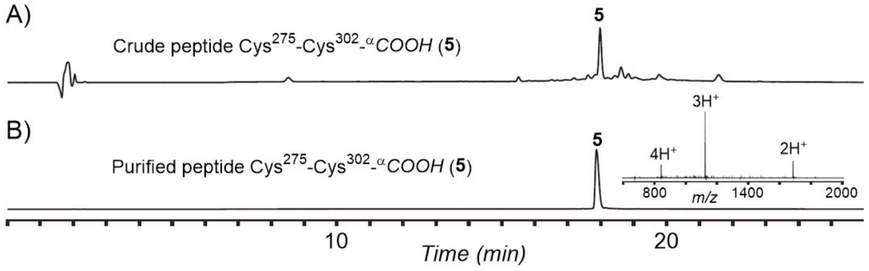

Figure S9. Analytical HPLC profile (λ= 214 nm) together with ESI-MS data (inset) of Cys^{275}-Cys^{302}-$^{\alpha}$*COOH* (**5**). (A) Crude peptide **5**. (B) Purified peptide **5**. Linear gradient 10%-54% of B over 22 min including 4 min equilibration using Agilent Zorbax SB-C3, 5 µm, 4.6 × 150 mm, LC column with 0.9 mL/min flow rate was used for the chromatographic separation. Purification was performed using a linear gradient 15%-35% of buffer B in buffer A over 60 min with a flow rate of 5 mL/min at 40 °C (buffer A = 0.1% TFA in water; buffer B = 0.08% TFA in acetonitrile) using a C12, 10 × 250 mm column (Phenomenex proteo, 90 Å, 4 µm).

6.2. Synthesis of *Pf*-AMA1 (3D7 strain) peptide segment *Fmoc*-Cys^{247}-Phe^{274}-$^{\alpha}$*COSR'* (6)

The peptide *Fmoc*-Cys-His-Ile-Leu-Tyr-Ile-Ala-Ala-Gln-Glu-Asn-Asn-Gly-Pro-Arg-Tyr-Cys-Asn-Lys-Asp-Glu-Ser-Lys-Arg-Asn-Ser-Met-Phe-$^{\alpha}$*CONHNH$_2$* (**6'**) was synthesized using NH_2NH-2-Cl-(Trt)-resin (scale = 0.3 mmol; substitution = 0.672 mmol/g) by stepwise Fmoc chemistry SPPS in automated peptide synthesizer at 50 °C (see *Section-1.3* for the peptide synthesis protocol). After global deprotection using TFA cocktail, the crude peptide **6'** was precipitated using diethyl ether. The mass of the peptide **6'** was confirmed by LC-MS (**Figure S10A,** Observed mass (ESI-MS): 3538.63 Da (deconvoluted most abundant isotopologue); calculated mass: 3538.63 Da (most abundant isotopologue). The crude peptide *Fmoc*-Cys^{247}-Phe^{274}-$^{\alpha}$*CONHNH$_2$* (**6',** ~0.2 mmol) was then dissolved in 20 mL of aqueous phosphate buffer (0.2 M) containing 6 M Gu.HCl at pH 3.0 and kept at -16.5 °C (Julabo). After 15 min, 2 mL aqueous $NaNO_2$ (0.5 M) solution was added to the solution of peptide **6'** and gently agitated for 15 min at -16.5 °C. Then, 13.5 mL of 0.3 M MESNa containing 0.2 M aqueous phosphate buffer and 6 M Gu.HCl at pH 6.2 was mixed into the oxidized solution of peptide **6'** and the

temperature was raised to RT. The pH was adjusted to 5.1. The MESNa exchange was essentially complete within 20 min as monitored by LCMS (**Figure S10B**). Afterward, 520 mg of TCEP (50 mM) was added to the reaction mixture as a solid powder. The pH of the resulting reaction mixture was adjusted to 3.6 and agitated for further 30 min (Note: thiolactone formation (-32 Da from parent peptide hydrazide) was observed at pH higher than 4.5; therefore, the reaction mixture should be allowed to incubate at pH <4 for longer time in case of incomplete disulfide reduction). Purification by preparative HPLC gave 112.9 mg (31 µmol, 15.5%) of the desired peptide segment *Fmoc*-Cys^{247}-Phe^{274}–$^{\alpha}$*COSR′* (**6**). Observed mass (ESI-MS): 3648.56 Da (deconvoluted most abundant isotopologue); calculated mass: 3648.56 Da (most abundant isotopologue) (**Figure S10C**).

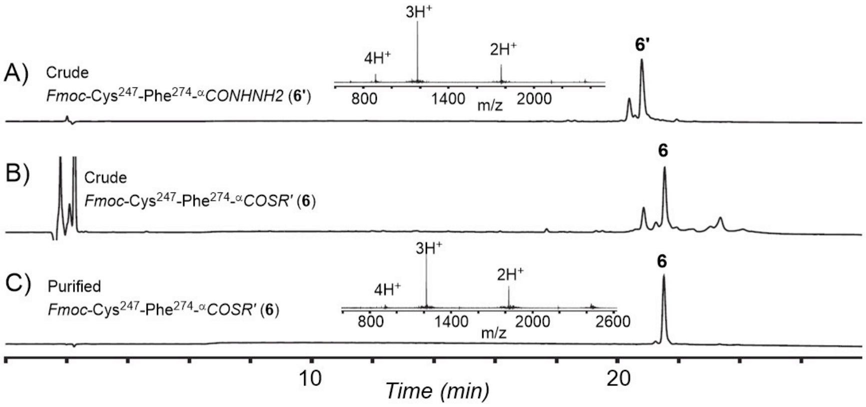

Figure S10. Analytical HPLC profile (λ= 214 nm) together with ESI-MS data (inset). (A) Chromatogram of the crude *Fmoc*-Cys^{247}-Phe^{274}-$^{\alpha}$*CONHNH$_2$* peptide (**6'**). (B) Chromatogram of the crude *Fmoc*-Cys^{247}-Phe^{274}-$^{\alpha}$*CONHNH$_2$* after $NaNO_2$ mediated oxidation and MESNa exchange. (C) Chromatogram of the purified peptide *Fmoc*-Cys^{247}-Phe^{274}- $^{\alpha}$*COSR′* (**6**). R′ = *–$CH_2CH_2SO_3Na$*. Linear gradient 10%-54% of B over 22 min including 4 min equilibration using an Agilent Zorbax SB-C3, 5 µm, 4.6 × 150 mm LC column with 0.9 mL/min flow rate was used for the chromatographic separation. Purification was performed using a linear gradient 20%-40% of buffer B in buffer A over 40 min with a flow rate of 5 mL/min at 40 °C (buffer A = 0.1% TFA in water; buffer B = 0.08% TFA in acetonitrile) using an Agilent ZORBAX-SB, 5 µm, (C3), 9.4 × 250 mm LC column.

6.3. Synthesis of *Pf*-AMA1 (3D7) peptide segment *Fmoc*-Cys^{217}-Lys^{246}- $^{\alpha}$*COSR′* (7)

The peptide *Fmoc*-Cys-Ser-Arg-His-Ala-Gly-Asn-Met-Ile-Pro-Asp-Asn-Asp-Lys-Asn-Ser-Asn-Tyr-Lys-Tyr-Pro-Ala-Val-Tyr-Asp-Asp-Lys-Asp-Lys-Lys-$^{\alpha}$*CONHNH$_2$* (**7'**) was synthesized using NH_2-NH-2-Cl-(Trt)-resin (scale = 0.3 mmol; substitution = 0.683 mmol/g) by stepwise Fmoc chemistry SPPS in an automated peptide synthesizer at 50 °C (*see* **Section-1.3** for the peptide synthesis protocol). After global deprotection using TFA cocktail, the crude peptide **7'** was precipitated using diethyl ether. The purity and the mass of the peptide **7'** was confirmed by LC-MS (**Figure S11-A).** Observed mass (**ESI-MS**): 3723.72 Da (deconvoluted most abundant isotopologue); calculated mass: 3723.72 Da (most abundant isotopologue). Crude peptide *Fmoc*-Cys^{217}-Lys^{246}- $^{\alpha}$*CONHNH$_2$* **7' (**~500 mg, ~0.15 mmol) was then dissolved in 20 mL of aqueous phosphate buffer (0.2 M) containing 6 M Gu.HCl at pH 3.0 and kept at -16.5 °C (Julabo). After 15 min, 2 mL aqueous $NaNO_2$ (0.5 M) solution was added to the solution of peptide **7'** and gently agitated for 15 min at -16.5 °C. Then, 13.5 mL of 0.3 M MESNa in pH 6.3 buffer (200 mM PB, 6 M Gu.HCl) was mixed into the oxidized solution of peptide **7'** and the temperature was raised to RT. The pH was adjusted to 5.0 and incubated for 30 min to afford the MESNa exchanged product as monitored by LCMS (**Figure S11-B)**. Afterward, 510 mg of TCEP (50 mM) was added as solid to the reaction mixture, pH was adjusted to 3.5, and the reaction mixture was agitated for further 30 min (Note: thiolactone formation (-32 Da from the parent peptide hydrazide) was observed at pH higher than 4.5; therefore, the reaction mixture should be allowed to incubate at pH <4 for longer time in case of incomplete disulfide reduction). Purification by preparative HPLC furnished 156.7 mg (41 µmol, 27.3%) of the purified peptide segment *Fmoc*-Cys^{217}-Lys^{246}-$^{\alpha}$*COSR′* (**7**). Observed mass (ESI-MS): 3833.65 Da (deconvoluted most abundant isotopologue); calculated mass: 3833.65 Da (most abundant isotopologue) (**Figure S11-C**).

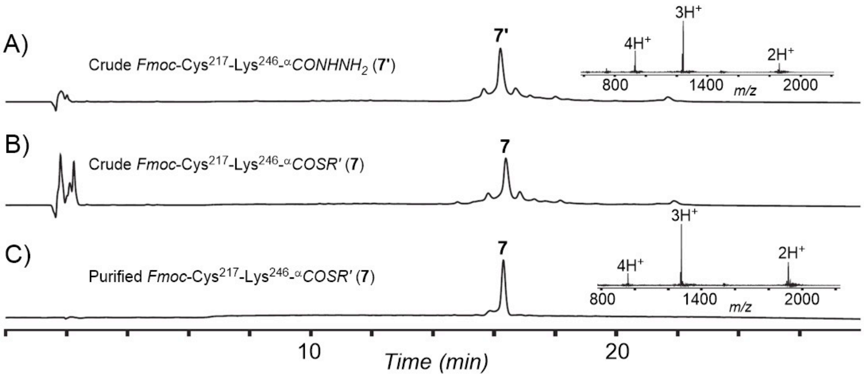

Figure S11. Analytical HPLC profile (λ= 214 nm) together with ESI-MS data (inset). (A) Chromatogram of the crude *Fmoc*-Cys^{217}-Lys^{246}-$^{\alpha}$*CONHNH₂* peptide (**7'**) (B) Chromatogram of the crude *Fmoc*-Cys^{217}-Lys^{246}-$^{\alpha}$*CONHNH₂* after $NaNO_2$ mediated oxidation and MESNa exchange (C) Purified peptide *Fmoc*-Cys^{217}-Lys^{246}-$^{\alpha}$*COSR'* (**7**). *R'* = $-CH_2CH_2SO_3Na$. Linear gradient 10%-54% of B over 22 min including 4 min equilibration using Agilent Zorbax SB-C3 5 µm 4.6 × 150 mm LC column with 0.9 mL/min flow rate was used for the chromatographic separation. Purification was performed using a linear gradient 10%-30% of buffer B in buffer A over 60 min with a flow rate of 5 mL/min at 40 °C (buffer A = 0.1% TFA in water; buffer B = 0.08% TFA in acetonitrile) using an Agilent ZORBAX-SB, 5 µm (C3), 9.4 × 250 mm LC column.

6.4. One-Pot multi-segment synthesis of *Pf*-AMA1-polypeptide Cys^{217}-Cys^{302}- $^{\alpha}$*COOH* (11)

For the first ligation, the peptide segment Cys^{275}-Cys^{302}-$^{\alpha}$*COOH* (**5**, 10 mg, 2.9 µmol) was dissolved in 800 µL degassed ligation buffer (200 mM phosphate buffer, 6 M Gu.HCl and 100 mM TCEP) containing 100 mM MPAA, and the buffer pH was adjusted to 6.8. Next, the peptide segment *Fmoc*-Cys^{247}-Phe^{274}- $^{\alpha}$*COSR'* (**6**, 11.9 mg, 3.26 µmol) was added to the reaction mixture as a solid powder. The pH of the reaction mixture was then readjusted to 6.8 and was allowed to react at room temperature to furnish the ligation product *Fmoc*-Cys^{247}-Cys^{302}-COOH (**8**) within 24 h (**Figure 3C-b, main text**). To remove the Fmoc group of the ligated product **8** after the first ligation, ~65 µL concentrated HCl was added followed by ~220 µL piperidine to the reaction mixture to prevent a sudden abrupt change in pH and to fix the final concentration of piperidine to 20% (v/v). The final pH of the reaction mixture was immediately adjusted to 11.0 by using concentrated HCl (12 M) and aqueous NaOH (6 M). The reaction mixture was vortexed vigorously at room temperature. The complete Fmoc deprotection was observed within 7 min (including ~2 min time required for pH adjustment) to afford Cys^{247}-Cys^{302}-$^{\alpha}$*COOH* (**9**) (**Figure 3C-c, main text**). The pH of the reaction mixture was then rapidly brought down to ~9 by adding concentrated HCl (12 M) followed by the addition of TCEP as a solid powder (final concentration 50 mM). The pH of the reaction mixture was then adjusted to 6.90. For the second ligation, peptide segment *Fmoc*-Cys^{217}-Lys^{246}-$^{\alpha}$*COSR'* (**7**, 13.65 mg, 3.56 µmol) was then added to the reaction mixture and the pH was adjusted back to 6.85. Within 20 h, the peptide **9** was completely converted to the desired ligation product *Fmoc*-Cys^{217}-Cys^{302}-$^{\alpha}$*COOH* (**10**) (**Figure 3C-e, main text**). To deprotect the Fmoc group from peptide **10**, additional piperidine (to adjust the total concentration to 20% (v/v)) and 6 M NaOH (to increase the pH to 11.0) were added to the reaction mixture. The reaction mixture was then vortexed for a total of 7 min (including ~2 min time required for adjusting pH) to afford the final polypeptide **11** (**Figure 3C-f, main text**). As before, the pH of the reaction buffer was rapidly reduced to ~9.0 by adding concentrated HCl followed by the addition of TCEP (final concentration 50 mM) as solid powder, and the pH was readjusted to 7.0 to achieve complete disulfide reduction. After the two ligations, and two Fmoc deprotection steps, HPLC purification was performed to obtain the pure full-length polypeptide Cys^{217}-Cys^{302}-$^{\alpha}$*COOH* (**11**, 10.9 mg, 1.08 µmol) with an overall 37% yield. The purity and identity of the *Pf*-AMA1 polypeptide **11** were confirmed by LC-MS (**Figure 3C-g & Figure 3C-h, main text**). Observed mass (**ESI-MS**): 10123.28 ± 0.06 Da (average of the eight most abundant charge states); calculated mass: 10123.21 Da (average isotope composition).

7. One-pot total chemical synthesis of human lysozyme

7.1. Synthesis of lysozyme segment Cys^{95}-Val^{130}- $^{\alpha}$*COOH* (12)

The peptide Cys-Ala-Lys-Arg-Val-Val-Arg-Asp-Pro-Gln-Gly-Ile-Arg-Ala-Trp-Val-Ala-Trp-Arg-Asn-Arg-Cys-Gln-Asn-Arg-Asp-Val-Arg-Gln-Tyr-Val-Gln-Gly-Cys-Gly-Val-$^{\alpha}$*COOH* (**12**) was synthesized on 2-Cl-(Trt)-Cl resin (substitution = 0.143 mmol/g) in an

automated peptide synthesizer (**see *Section-1.3*** for the peptide synthesis protocol) on a 0.2 mmol scale. Global deprotection and the cleavage of the peptide from the resin using a TFA cocktail followed by HPLC purification afforded 217 mg (25.56 mmol, 25.6%) of the desired peptide **12,** Cys^{95}-Val^{130}-$^{\alpha}$*COOH* (**Figure S17**). Observed mass (**ESI-MS**): 4244.17 Da (deconvoluted most abundant isotopologue); calculated mass: 4244.17 Da (most abundant isotopologue).

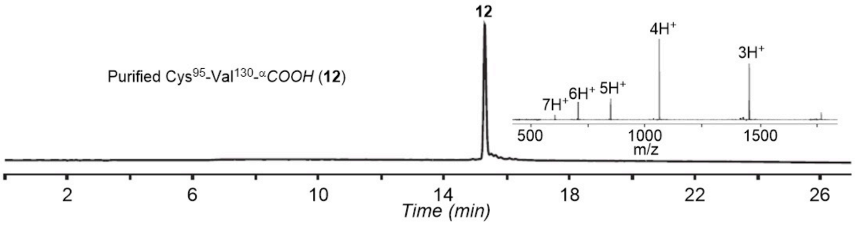

Figure S17. Analytical RP-HPLC profile (λ = 214 nm) together with ESI-MS data (inset) of the purified peptide Cys^{95}-Val^{130}- $^{\alpha}$*COOH* (**12**) synthesized on 2-Cl-(Trt)-Cl resin. Linear gradient 10-54% of B over 22 min including 4 min equilibration using Agilent Zorbax SB-C3, 5µm, 4.6 × 150mm LC column with 0.9 mL/min flow rate was used for the chromatographic separation. Purification was performed using a linear gradient 25%-45% buffer B in buffer A over 40 min with a flow rate of 5 mL/min at 40 °C (buffer A = 0.1% TFA in water; buffer B = 0.08% TFA in acetonitrile) using a C4, 10 × 250 mm column (Phenomenex proteo, 300 Å, 10 µm).

7.2. Synthesis of lysozyme segment *Fmoc*-Cys^{65}-Ala^{94}-$^{\alpha}$*COSR'* (13)

The peptide with the sequence *Fmoc*-Cys-Asn-Asp-Gly-Lys-Thr-Pro-Gly-Ala-Val-Asn-Ala-Cys-His-Leu-Ser-Cys-Ser-Ala-Leu-Leu-Gln-Asp-Asn-Ile-Ala-Asp-Ala-Val-Ala-$^{\alpha}$*COSR'* (**13**) was prepared from the corresponding peptide hydrazide **13' (Figure S18)**. The hydrazide peptide *Fmoc*-Cys^{65}-Ala^{94}- $^{\alpha}$*CONHNH$_2$* (**13'**) was synthesized on NH_2NH-2-Cl-(Trt)-resin (substitution = 0.41 mmol/g) by stepwise Fmoc chemistry SPPS on a 0.2 mmol scale in an automated peptide synthesizer (**see *Section-1.3*** for the peptide synthesis protocol). After the global deprotection using TFA cocktail, the crude peptide **13'** was precipitated using diethyl ether. The purification of the crude peptide by preparative HPLC afforded 173 mg (41.98 µmol, 27%) of the pure peptide **13' (Figure S18-a**). Observed mass (ESI-MS): 3207.46 Da (deconvoluted most abundant isotopologue); calculated mass: 3207.46 Da (most abundant isotopologue). The purified peptide **13'** (100 mg, 31.16 µmol) was then dissolved in 20 mL of aqueous phosphate buffer (0.2 M) containing 6 M Gu.HCl at pH 3.0 and kept at -16.5 °C (Julabo). After 15 min, 2.0 mL aqueous $NaNO_2$ (0.5 M) was added to the solution of peptide **13'** and gently agitated for 15 min at -16.5 °C. Then, 20 mL of 0.2 M MESNa containing 0.2 M aqueous phosphate buffer and 6 M Gu.HCl at pH 6.15 was mixed into the oxidized solution of peptide **13'** and the temperature was allowed to rise to RT. The pH of the reaction mixture was then adjusted to 6.1, and the MESNa exchange was complete within 30 min as observed from LCMS **(Figure S18-b**). Afterward, 602 mg of TCEP (50 mM) was added to the reaction mixture as a solid powder. The pH of the resulting reaction mixture was adjusted to 3.4 and agitated further for 30 min. Purification of the crude reaction mixture by preparative HPLC gave 42.39 mg (12.77 µmol, 41% yield) of the desired MESNa exchanged peptide **13 (***Fmoc*-Cys^{65}-Ala^{94}-$^{\alpha}$*COSR'*, **Figure S18-c**). Observed mass (**ESI-MS**):3318.40 Da (deconvoluted most abundant isotopologue); calculated mass: 3318.39 Da (most abundant isotopologue).

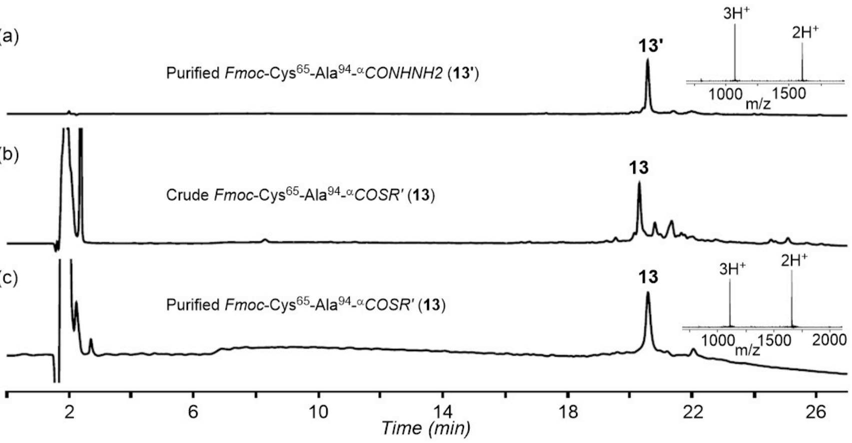

Figure S18. Analytical RP-HPLC profile (λ = 214 nm) together with ESI-MS data (inset) for the synthesis of peptide *Fmoc*-Cys[65]-Ala[94]-$^{\alpha}$*COSR'* (**13**). *R' = –CH₂CH₂SO₃Na*. (A) Chromatogram of the crude peptide *Fmoc*-Cys[65]-Ala[94]-$^{\alpha}$*CONHNH₂* (**13'**) (B) Chromatogram for the crude MESNa exchanged product *Fmoc*-Cys[65]-Ala[94]-$^{\alpha}$*COSR'* (**13**). (C) Chromatogram for the purified peptide MESNa exchanged product *Fmoc*-Cys[65]-Ala[94]-$^{\alpha}$*COSR'* (**13**). Linear gradient 10%-54% of B over 22 min including 4 min equilibration using Agilent Zorbax SB-C3, 5µm, 4.6 × 150mm LC column with 0.9 mL/min flow rate was used for all the chromatographic separation. Purification of both the peptides were performed using a linear gradient 25%-45% buffer B in buffer A over 60 min with a flow rate of 5 mL/min at 40 °C (buffer A = 0.1% TFA in water; buffer B = 0.08% TFA in acetonitrile) using a C4, 10 × 250 mm column (Phenomenex proteo, 300 Å, 10 µm).

7.3. Synthesis of lysozyme segment *Fmoc*-Cys[30]-Trp[64]-$^{\alpha}$*COSR'* (14a)

The peptide with the sequence *Fmoc*-Cys-Leu-Ala-Lys-Trp-Glu-Ser-Gly-Tyr-Asn-Thr-Arg-Ala-Thr-Asn-Tyr-Asn-Ala-Gly-Asp-Arg-Ser-Thr-Asp-Tyr-Gly-Ile-Phe-Gln-Ile-Asn-Ser-Arg-Tyr-Trp-$^{\alpha}$*COSR'* (**14a**) was prepared from the corresponding peptide hydrazide **14' (Figure S19).** The peptide hydrazide **14'** was synthesized on NH_2NH-2-Cl-(Trt)-resin (substitution = 0.44 mmol/g) by stepwise Fmoc chemistry SPPS in 0.2 mmol scale in an automated peptide synthesizer (see **Section-1.3** for the peptide synthesis protocol). After the global deprotection using a TFA cocktail, the crude peptide **14'** was precipitated using diethyl ether and purified by preparative HPLC to afford 183 mg (41.98 µmol, 21%) of the pure peptide **14' (Figure S19-A**). Observed mass (ESI-MS): 4357.99 Da (deconvoluted most abundant isotopologue); calculated mass: 4357.99 Da (most abundant isotopologue). Purified peptide **14'** (120 mg, 27.53 µmol) was dissolved in 25 mL of aqueous phosphate buffer (0.2 M) containing 6 M Gu.HCl at pH 3.0 and incubated at -16.5 °C (Julabo). After 15 min, 2.5 mL aqueous $NaNO_2$ (0.5 M) solution was added to the solution of peptide **14'** and gently agitated for 15 min at -17 °C. Then, 25 mL of 0.2 M MESNa containing 0.2 M aqueous phosphate buffer and 6 M Gu.HCl at pH 6.15 was mixed into the oxidized solution of peptide **14'** and the temperature was allowed to rise to RT. The pH was adjusted to 6 and the MESNa exchange was complete within 20 min (monitored by LCMS). Afterward, TCEP (751.5 mg, 50 mM) was added to the reaction mixture as a solid powder. The pH of the resulting reaction mixture was adjusted to 3.25 and agitated further for 30 min (Note: Incubation at pH higher than 4.5 produced thiolactone (-32 Da from the parent peptide hydrazide mass); hence, the reaction mixture should be agitated at pH <4 for a longer time in case of incomplete disulfide reduction). Purification by preparative HPLC furnished 52.89 mg (11.83 µmol, 43% yield) of the desired MESNa exchanged peptide **14a** (*Fmoc*-Cys[30]-Trp[64]-$^{\alpha}$*COSR'*, **Figure S19-B**). Observed mass (**ESI-MS**): 4467.92 Da (deconvoluted most abundant isotopologue); calculated mass: 4467.93 Da (most abundant isotopologue).

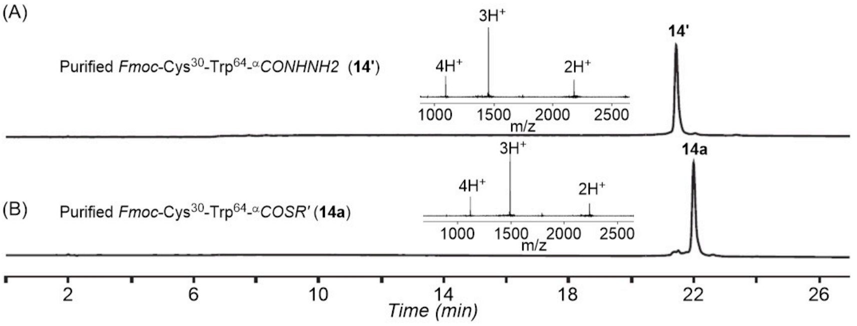

Figure S19. Analytical RP-HPLC profile (λ = 214 nm) together with ESI-MS data (inset) for the synthesis of peptide *Fmoc*-Cys[30]-Trp[64]-$^{\alpha}$*COSR'* (**14a**). *R' = –CH₂CH₂SO₃Na.* (A) Chromatogram of the pure peptide *Fmoc*-Cys[30]-Trp[64]-$^{\alpha}$*CONHNH₂* (**14'**). (B) Chromatogram of the pure MESNa exchanged product Fmoc-Cys[30]-Trp[64]-$^{\alpha}$*COSR'* (**14a**). Linear gradient 10%-54% of B over 22 min including 4 min equilibration using Agilent Zorbax SB-C3, 5µm, 4.6 × 150mm LC column with 0.9 mL/min flow rate was used for all the chromatographic separations. Purification of both the peptides were performed using a linear gradient 25%-45% buffer B in buffer A over 60 min with a flow rate of 5 mL/min at 40 °C (buffer A = 0.1% TFA in water; buffer B = 0.08% TFA in acetonitrile) using a C4, 10 × 250 mm column (Phenomenex proteo, 300 Å, 10 µm).

7.4. Synthesis of lysozyme segment Lys[1]-Met[29]-$^{\alpha}$*COSR'* (15)

The peptide with the sequence Lys-Val-Phe-Glu-Arg-Cys-Glu-Leu-Ala-Arg-Thr-Leu-Lys-Arg-Leu-Gly-Met-Asp-Gly-Tyr-Arg-Gly-Ile-Ser-Leu-Ala-Asn-Trp-Met-$^{\alpha}$*COSR'* (**15**) was prepared from the corresponding hydrazide peptide **15'** (**Figure S20**). The peptide hydrazide **15'** was synthesized on NH_2NH-2-Cl-(Trt)-resin (substitution = 0.34 mmol/g) by stepwise Fmoc chemistry SPPS on a 0.2 mmol scale in automated peptide synthesizer (see **Section-1.3** for the peptide synthesis protocol). After the global deprotection of the peptide using a TFA cocktail, the crude peptide **15'** was precipitated using diethyl ether and the purification using preparative HPLC gave 157 mg (45.78

µmol, 22.9%) of pure peptide **15'** (**Figure S20-A).** Observed mass (**ESI-MS**): 3428.80 Da (deconvoluted most abundant isotopologue); calculated mass: 3428.80 Da (most abundant isotopologue). Purified peptide **15'** (100 mg, 29.16 µmol) was dissolved in 15 mL of aqueous phosphate buffer (0.2 M) containing 6 M Gu.HCl at pH 3.0 and incubated at -16.5 °C (Julabo). After 15 min, 1.5 mL aqueous $NaNO_2$ (0.5 M) solution was added to the solution of peptide **15'** and gently agitated for 15 min at -16.5 °C. Then, 15 mL of 0.2 M MESNa containing 0.2 M aqueous phosphate buffer and 6 M Gu.HCl at pH 6.30 was mixed into the oxidized solution of peptide **15'** and the temperature was raised to room temperature. The pH was adjusted to 6.1 and the MESNa exchange was complete within 20 min (monitored by LCMS). Afterward, 450 mg of TCEP (~50 mM) was added as a solid powder. The pH of the resulting reaction mixture was adjusted to 3.5 and gently agitated for 30 min (Note: Incubation at pH higher than 4.5 produced thiolactone (-32 Da from the peptide hydrazide mass); hence, the reaction mixture should be agitated at pH <4 for a longer time in case of incomplete disulfide reduction). Purification using preparative HPLC furnished 64 mg (18.08 µmol, 62% yield) of the desired MESNa exchanged peptide **15,** Lys^1-Met^{29}-$^{\alpha}$*COSR'* (**Figure S20-B**). Observed mass (**ESI-MS**): 3538.74 Da (deconvoluted most abundant isotopologue); calculated mass: 3538.74 Da (most abundant isotopologue).

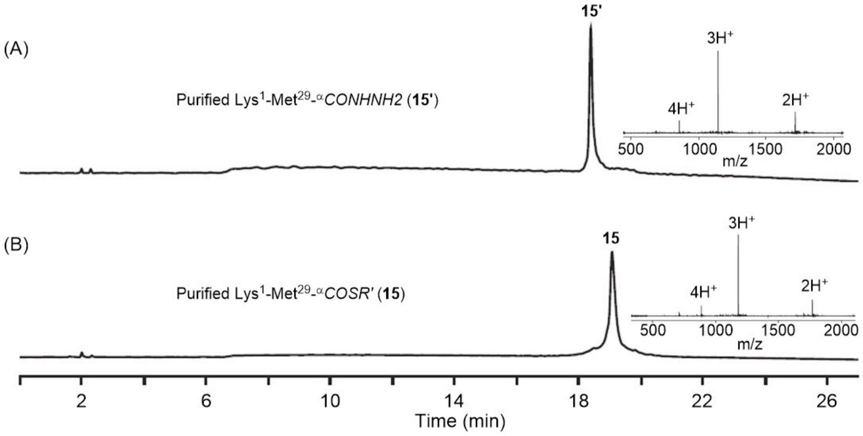

Figure S20. Analytical RP-HPLC profile (λ = 214 nm) together with ESI-MS data (inset) for the synthesis of peptide Lys^1-Met^{29}-$^{\alpha}$*COSR'* (**15**). (A) Chromatogram for the pure peptide Lys^1-Met^{29}-$^{\alpha}$*CONHNH$_2$* (**15'**). (B) Chromatogram for the pure MESNa exchanged product Lys^1-Met^{29}-$^{\alpha}$*COSR'* (**15**). *R' = $-CH_2CH_2SO_3Na$.* Linear gradient 10%-54% of B over 22 min including 4 min equilibration using Agilent Zorbax SB-C3, 5µm, 4.6 × 150mm LC column with 0.9 mL/min flow rate was used for all the chromatographic separation. Purification of both the peptides were performed using a linear gradient of 15%-35% buffer B in buffer A over 60 min with a flow rate of 5 mL/min at 40 °C (buffer A = 0.1% TFA in water; buffer B = 0.08% TFA in acetonitrile) using a C4, 10 × 250 mm column (Phenomenex proteo, 300 Å, 10 µm).

7.5. One-pot four-segment ligation of human lysozyme peptide segment 12, 13, 14a and 15

To a solution of peptide **12** (30 mg, 7.06 µmol) in 15 mL of the ligation buffer (200 mM PB, 6 M Gu.HCl, 20 mm TCEP) containing 50 mM MPAA was added peptide **13** (23.42 mg, 7.06 µmol) and the pH of the reaction mixture was adjusted to 6.7. The first ligation was completed in 36 h to yield the ligated product **16**. Then, piperidine was added to the reaction mixture and the pH was adjusted to 11.0 by adding concentrated HCl maintaining the final concentration of piperidine to 20% (v/v) to deprotect the Fmoc group from the ligated product **16** to yield peptide **17**. Immediately after 7 min incubation, the pH of the reaction mixture was rapidly reduced to 9.0 by adding concentrated HCl. Solid TCEP (104 mg, ~20 mM) was then added and the pH was readjusted to 7.0. The third peptide segment **14a** (31.54 mg, 7.06 µmol) was then added to the reaction mixture and the pH was readjusted to 6.7. The second ligation reaction was essentially completed within 30 h. To yield the Fmoc deprotected peptide **19** from the ligated product **18**, the pH was raised to 11.0 by adding 6M NaOH maintaining the final piperidine concentration to 20% (v/v). After 7 min, the pH of the reaction mixture was rapidly reduced to 7.0 by adding concentrated HCl followed by solid TCEP (104 mg, ~20 mM). After 5 min, the last peptide segment **15** (25 mg, 7.06 µmol) was added to the same reaction mixture and the pH was readjusted to 6.7. The third and the final ligation was completed within 30 h furnishing the full-length lysozyme polypeptide **20**. The one-pot four-segment ligation sequences were monitored by analytical HPLC as shown in **Figure S21**. The purification of the full-length polypeptide by preparative HPLC afforded 37.40 mg (2.54 µmol, 36% yield based on the starting peptide segment **12**) of the pure desired peptide **20** (Observed mass (**ESI-MS**): 14700.70 ± 0.04 Da Da (average deconvoluted isotope composition); calculated mass: 14700.63 Da (average isotope composition)).

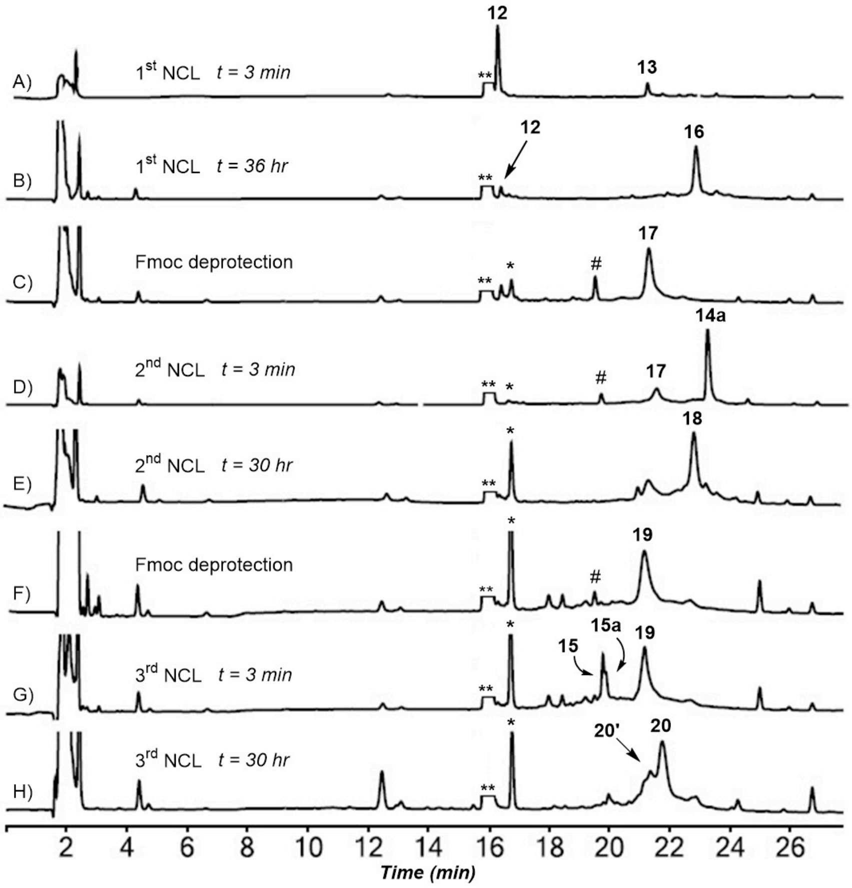

Figure S21. Analytical HPLC profile (λ= 214 nm) of the one-pot synthesis of the lysozyme polypeptide. (A) Chromatogram at 3 min after addition of peptide **12** and **13** in ligation buffer (200 mM phosphate, 6 M Gu.HCl, 20 mM TCEP) containing 50 mM MPAA. (B) The first ligation was completed within 36 h and gave **16** as the ligated product. (C) Fmoc deprotection from the ligated product **16** to obtain peptide **17**. (D) Chromatogram at 3 min after the addition of peptide **14a** in the reaction mixture. (E) The second ligation was essentially complete within 30 h furnishing the polypeptide **18**. (F) Fmoc deprotection of the ligated product **18** to get peptide **19**. (G) Chromatogram at 3 min after the addition of peptide **15** in the same reaction mixture. **15a** is the MPAA exchanged product from peptide **15**. (H) The third ligation was completed within 30 h and afforded polypeptide **20**. The side product peaks, obtained from the thiolactone of excess segment **15** and from the ligation between the left out **15** and **14a**, were clustered (**20'**) and eluted in the RP-HPLC column just before the full-length polypeptide peak **20**. Purification was carried out on an Agilent zorbax SB-C3 5 µm, 9.4 × 250 mm, LC column using a linear gradient of 20%-40% of buffer B over 60 min with a flow rate of 5 mL/min at 40 °C. The '**' indicates MPAA, '#' indicates piperidine adduct with dibenzofulvene and '*' indicates the TCEP adduct with dibenzofulvene.

8. Convergent synthesis of human lysozyme

8.1. Synthesis of lysozyme segment Cys^{30}-Trp^{64}-$^{\alpha}$*CONHNH$_2$* (14b)

The peptide with the sequence Cys-Leu-Ala-Lys-Trp-Glu-Ser-Gly-Tyr-Asn-Thr-Arg-Ala-Thr-Asn-Tyr-Asn-Ala-Gly-Asp-Arg-Ser-Thr-Asp-Tyr-Gly-Ile-Phe-Gln-Ile-Asn-Ser-Arg-Tyr-Trp-$^{\alpha}$*CONHNH$_2$* (**14b**) was prepared from the on-resin deprotection of N-terminal Fmoc group from **14'.** The synthesis of peptide **14'** was discussed in the previous section. After the global deprotection using a TFA cocktail, the crude peptide **14b** was precipitated using diethyl ether and purified by preparative HPLC to afford 183 mg (41.98 µmol, 21%) of the pure peptide **14b (Figure S22**). Observed mass (ESI-MS): 4135.94 Da (deconvoluted most abundant isotopologue); calculated mass: 4135.93 Da (most abundant isotopologue).

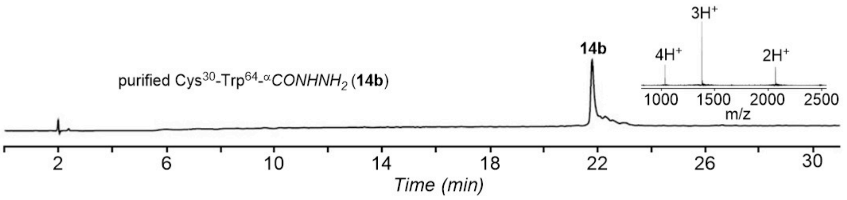

Figure S22. Analytical RP-HPLC profile (λ = 214 nm) together with ESI-MS data (inset) of the purified peptide Cys^{30}-Trp^{64}- $^{\alpha}$*CONHNH$_2$* (**14b**) synthesized on 2-Cl-(Trt) resin. Linear gradient 10-64% of B over 27 min including 4 min equilibration using Agilent Zorbax SB-C3, 5µm, 4.6x150mm LC column with 0.9 mL/min flow rate was used for the chromatographic separation. Purification was performed using a linear gradient 25%-45% buffer B in buffer A over 40 min with a flow rate of 5 mL/min at 40 °C (buffer A = 0.1% TFA in water; buffer B = 0.08% TFA in acetonitrile) using a C4, 10 × 250 mm column (Phenomenex proteo, 300 Å, 10 µm).

8.2. Four segment convergent synthesis of human lysozyme from peptide segment 12, 13, 14b and 15

For the synthesis of the left-hand half of the full-length lysozyme polypeptide, the peptide segment **14b** (14.8 mg, 3.57 µmol) and **15** (12.63 mg, 3.57 µmol) were mixed in 8 mL of the ligation buffer (200 mM PB, 6 M Gu.HCl, 20 mm TCEP) containing 50 mM MPAA and the pH was adjusted to 6.7. The ligation reaction was essentially completed within 30 h to yield the ligated product **21**. The ligation was monitored by analytical HPLC and ESI-MS as shown in **Figure S23**. The ligated product **21** was then purified using preparative HPLC to afford 15.06 mg (1.99 µmol, 56% yield based on the peptide segment **14b**) of the pure desired peptide **21** (Observed mass (**ESI-MS**): 7532.69 Da (deconvoluted most abundant isotopologue); calculated mass: 7532.69 Da (most abundant isotopologue)).

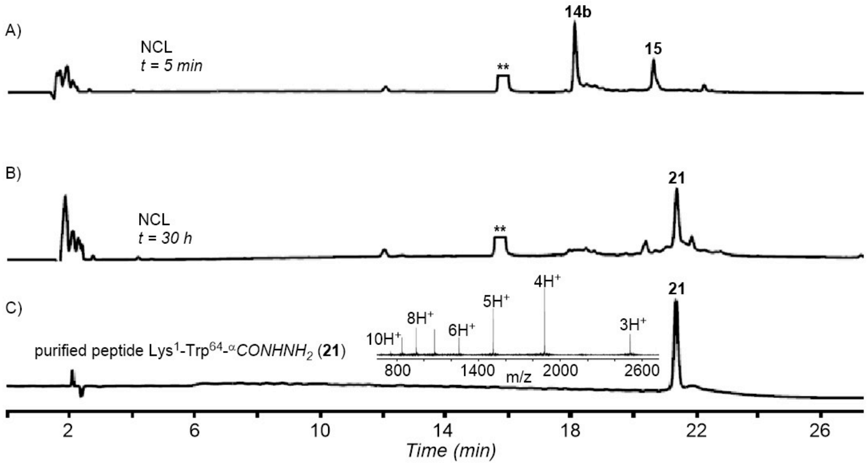

Figure S23. Analytical RP-HPLC profile (λ= 214 nm) together with ESI-MS data (inset) of the ligation reaction. (A) Chromatogram at 5 min after the addition of peptide Cys^{30}-Trp^{64}- $^{\alpha}$*CONHNH$_2$* **(14b)** and Lys^{1}-Met^{29}- $^{\alpha}$*COSR'* (**15**) in ligation buffer (200 mM phosphate, 6 M Gu.HCl, 20 mm TCEP) containing 20 mM MPAA. (B) Ligation was essentially completed within 30 h to yield ligated product **21**. (C) Chromatogram for the purified ligated peptide Lys^{1}-Trp^{64}- $^{\alpha}$*CONHNH$_2$* (**21**). Linear gradient 10%-54% of B over 22 min including 4 min equilibration using Agilent Zorbax SB-C3, 5 µm, 4.6 × 150 mm LC column with 0.9 mL/min flow rate was used for the chromatographic separation. Purification was carried out on an Agilent zorbax SB-C3 5 µm, 9.4 × 250 mm, LC column using a linear gradient of 20%-40% of buffer B over 60 min with a flow rate of 5 mL/min at 40 °C. The '**' indicates MPAA.

For the synthesis of the right-hand half of the full-length lysozyme polypeptide, to a solution of peptide **12** (11.71 mg, 3.53 µmol) in 7.0 mL of the ligation buffer (200 mM phosphate, 6 M Gu.HCl, 20 mm TCEP) containing 50 mM MPAA was added peptide **13** (15 mg, 3.53 µmol) and the pH of the reaction mixture was adjusted to 6.7. The first ligation was complete in 32 h furnishing peptide **16**. Then, piperidine was added to the reaction mixture and the pH was adjusted to 11.0 by adding concentrated HCl maintaining the final concentration of piperidine to 20% (v/v). Immediately after 7 min incubation, the pH of the reaction mixture was rapidly reduced to 9.0 by adding concentrated HCl. Solid TCEP (45 mg, ~20 mM) was then added and the pH was readjusted to 7.0. The ligation was monitored by analytical HPLC and ESI-MS as shown in **Figure S24**. The purification of the polypeptide **17** by preparative HPLC afforded 12.70 mg (1.76 µmol, 50% yield based on the peptide segment **12**) of the pure desired peptide **17** (Observed mass (**ESI-MS**): 7198.52 Da (deconvoluted most abundant isotopologue); calculated mass: 7198.52 Da (most abundant isotopologue)).

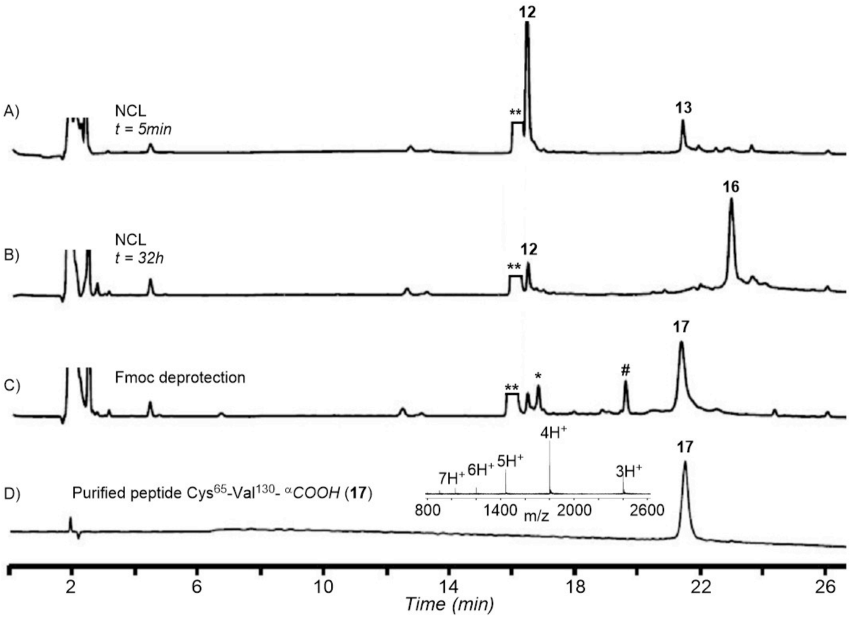

Figure S24. Analytical RP-HPLC profile (λ= 214 nm) together with ESI-MS data (inset) of the ligation reaction. (A) Chromatogram at 5 min after the addition of peptide Cys[95]-Val[130]-*αCOOH* **(12)** and *Fmoc*-Lys[65]-Ala[94]-*αCOSR'* **(13)** in ligation buffer (200 mM PB, 6 M Gu.HCl, 20 mm TCEP) containing 20 mM MPAA. (B) Ligation was essentially completed within 32 h to yield ligated product **16**. (C) Fmoc deprotection from ligated product **16** to get peptide Cys[65]-Val[130]-*αCOOH* (**17**). (D) Chromatogram for the purified ligated peptide Cys[65]-Val[130]-*αCOOH* (**17**). Linear gradient 10%-54% of B over 22 min including 4 min equilibration using Agilent Zorbax SB-C3, 5 µm, 4.6 × 150 mm LC column with 0.9 mL/min flow rate was used for the chromatographic separation. Purification was carried out on an Agilent zorbax SB-C3 5 µm, 9.4 × 250 mm, LC column using a linear gradient of 20%-40% of buffer B over 60 min with a flow rate of 5 mL/min at 40 °C. The '**' indicates MPAA, '*' indicates the TCEP adduct with dibenzofulvene and '#' indicates piperidine adduct with dibenzofulvene

To ligate the purified peptide **21** with **17**, the peptide **21** (10 mg, 1.33 µmol) was dissolved in 5 mL of aqueous phosphate buffer (0.2 M) containing 6 M Gu.HCl at pH 3.0 and incubated at -16.5 °C (Julabo). After 15 min, 500 µL aqueous $NaNO_2$ (0.5 M) solution was added to the solution of peptide **21** and gently agitated for 15 min at -17 °C. Then, 5 mL of MPAA (0.2 M) containing 0.2 M aqueous phosphate buffer and 6 M Gu.HCl at pH 6.30 was mixed into the oxidized solution of peptide **21**. Significant amount of thiolactone (**21'**) formation from the MPAA exchanged product **21''** was observed. Then the temperature was raised to room temperature and the peptide **17** (9.57 mg, 1.33 µmol) was added to the reaction mixture and the pH was readjusted to 6.7. The ligation was completed within 25 h furnishing the full-length lysozyme polypeptide **20**. The four-segment convergent ligation was monitored by analytical HPLC and ESI-MS as shown in **Figure S25**. The purification of the full-length polypeptide by preparative HPLC afforded 9.4 mg (0.64 µmol, 48% yield based on the limiting peptide segment **17**) of the pure desired peptide **20** (Observed mass (**ESI-MS**): 14700.72 ± 0.03 Da (average deconvoluted isotope composition); calculated mass: 14700.63 Da (average isotope composition)).

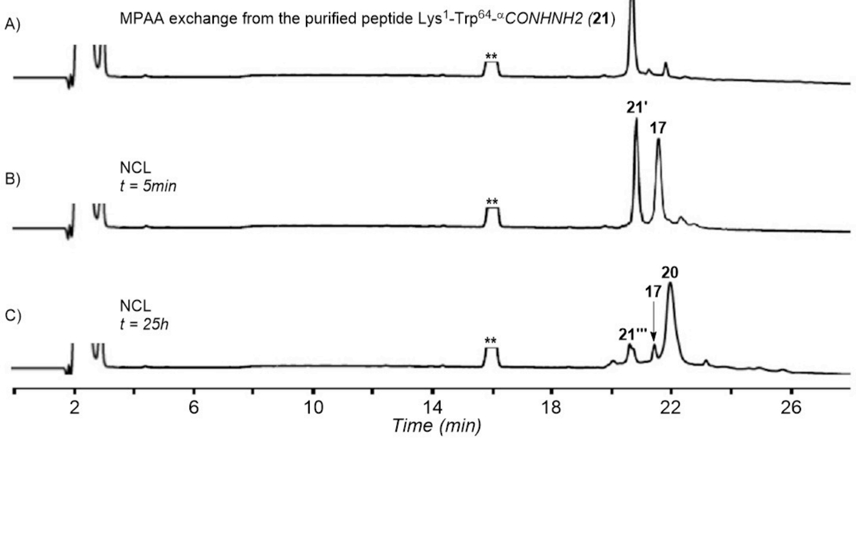

Figure S25. Analytical RP-HPLC profile (λ= 214 nm) together with ESI-MS data (inset) of the ligation reaction. (A) After $NaNO_2$ mediated oxidation and MPAA exchange of Lys^{1}-Trp^{64}-$^{\alpha}$*CONHNH$_2$* (**21**), significant amount of thiolactone (**21'**) formation from the MPAA exchanged product **21''** was observed. (B) Chromatogram at 5 min after the addition of peptide Cys^{65}-Val^{130}-$^{\alpha}$*COOH* (**17**). (C) Ligation was essentially completed within 25 h to yield ligated product **20**. A Tittle amount of hydrolysis product (**21'''**) from MPAA exchange product **21''** was observed. Linear gradient 10%-54% of B over 22 min including 4 min equilibration using Agilent Zorbax SB-C3, 5 µm, 4.6 × 150 mm LC column with 0.9 mL/min flow rate was used for the chromatographic separation. Purification was carried out on an Agilent zorbax SB-C3 5 µm, 9.4 × 250 mm, LC column using a linear gradient of 20%-40% of buffer B over 60 min with a flow rate of 5 mL/min at 40 °C. The '**' indicates MPAA.

9. Folding and characterization of chemically synthesized human lysozyme

9.1. Oxidative folding of lysozyme

The chemically synthesized full-length polypeptide **20** (15 mg, 1.02 µmol) was dissolved into 75 mL of the folding buffer containing a redox system consisting of 5 mM oxidized glutathione, 2 mM DTT in presence of 0.8 M Gu.HCl, and 1mM EDTA in 0.1M TRIS at pH 8. The folding with concomitant formation of four disulfide bonds was completed after 36 h, as determined by the LCMS. The folded lysozyme (**22**) eluted as a sharp peak with earlier retention time shift compared to the unfolded lysozyme and had a mass decrease of 7.6 Da, which was in a very good agreement with the formation of four disulfide bonds, revealed by ESI-MS (**Figure S26**). The purification of the folded lysozyme by HPLC furnished 5.70 mg (0.38 µmol, 38%) of the pure desired folded lysozyme molecule **22** (**Figure 4D, main text).** Observed mass (**ESI-MS**): 14692.71 ± 0.07 Da (average of the most abundant isotopologue mass over two observed charge states; calculated mass: 14692.56 Da (average isotope composition)).

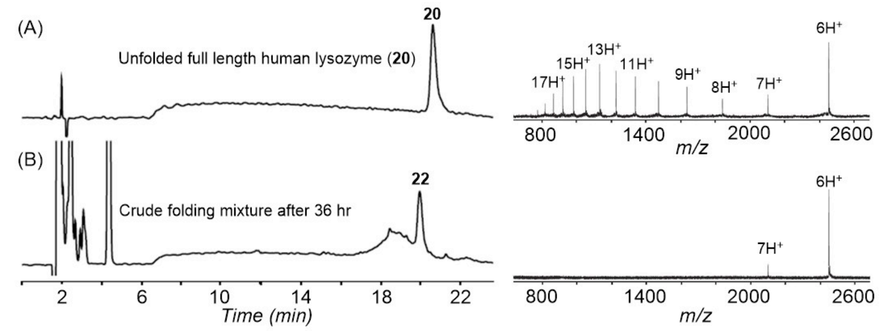

Figure S26. Analytical HPLC profile (λ= 214 nm) together with ESI-MS data (inset) for the oxidative folding of humane lysozyme. (A) Unfolded 130-residue lysozyme polypeptide (**20**). (B) After 36 h in folding buffer (0.1M TRIS-hydrochloride, 0.8M Gu.HCl, 5 mM oxidized glutathione, 2 mM DTT and 1mM EDTA at pH 8. The hump just before the correctly folded peak (**22**) in RP-HPLC indicates the accumulation of multiple misfolded isomers. Linear gradient 10%-54% of B over 22 min including 4 min equilibration time using Agilent Zorbax SB-C3, 5 µm, 4.6 × 150 mm LC column with 0.9 mL/min flow rate was used for the chromatographic separations. Purification of the folding reaction mixture was performed using a linear gradient 15%-45% buffer B in buffer A over 90 min with a flow rate of 5 mL/min using an Agilent ZORBAX-SB, 5 µm (C3), 9.4 × 250 mm LC column; where buffer A contains 0.1% TFA in water and B contains 0.08% TFA in acetonitrile**.**

9.2. Crystallization and X-ray structure determination of the synthetic human lysozyme

The chemically synthesized human lysozyme was crystallized following the reported crystallization conditions.[4] Typically, a 2 µL (14 mg/mL) solution of folded synthetic human lysozyme (**22**) in 120 mM LiCl, 2.5 mM HEPES, pH 7.5 was mixed with 2 µL of reservoir solution consisting of 30 mM sodium phosphate, 2.5M NaCl, pH 4.9. Crystals were grown at 24 °C using the hanging-drop vapor diffusion technique. Several diffraction quality crystals appeared within 4 days. X-ray datasets were collected at home source using marXperts Metal Jet (Excillum D2+) diffractometer equipped with liquid gallium anode and Pilatus 3R 1M detector. The X-ray data processing, scaling and merging were carried out using the software XDS.[5] A crystal that diffracted to 1.46 Å resolution was used for structure solution.

The structure of the synthetic lysozyme was solved by molecular replacement using PDB ID 2NWD[4] as the search model for phase determination using the program PHASER[6-7]. The structure was then refined using PHENIX REFINE. The final refined model had an R_{work}/R_{free} of 16.17%/19.57%. The data collection and the refinement statistics have been provided in Table S1. The final coordinates and the structure factors have been deposited in the protein data bank with the accession code 6LFH.

Table S1. X-ray data collection and refinement statistics of chemically synthesized human lysozyme.

Data collection*	
Space group	P 21 21 21
Wavelength (Å)	1.3417
Cell dimensions	
a, b, c (Å)	32.95 56.41 61.22
α, β, γ (°)	90.0, 90.0, 90.0
Mol/asymmetric unit	1
Mol/unit cell	4
Resolution (Å)	60.0 - 1.46 (1.50 – 1.46)
R_{merge}	0.048 (0.610)
CC1/2	0.999 (0.827)
$I/\sigma I$	29.68 (2.18)
Redundancy	9.2 (3.7)
Refinement*	
Resolution (Å)	30.6 - 1.46 (1.54 – 1.46)
No. reflections	19724
Completeness (%)	96.76 (81)
R_{work}/ R_{free}	0.162 (0.275) / 0.196 (0.294)
No. atoms	
Non-solvent	1104
Solvent	213
Average B-factor ($Å^2$)	19.24
RMS deviations	
Bond lengths (Å)	0.005
Bond angles (°)	0.77

*Highest resolution shell is shown in parentheses.

Coordinates and structure factors have been deposited in the Protein Data Bank with accession code 6LFH.

References

1. J. M. Collins, S. K. Singh, Coupling method for peptide synthesis at elevated temperatures. **2016**.
2. J. S. Zheng, S. Tang, Y. K. Qi, Z. P. Wang, L. Liu, Chemical synthesis of proteins using peptide hydrazides as thioester surrogates. *Nat. Protoc.* **2013,** *8*, 2483-2495.
3. S. H. Liu, B. L. Pentelute, S. B. H. Kent, Convergent Chemical Synthesis of [Lysine 24, 38, 83] Human Erythropoietin. *Angew. Chem., Int. Ed.* **2012,** *51*, 993-999.
4. T. Durek, V. Y. Torbeev, S. B. H. Kent, Convergent chemical synthesis and high-resolution x-ray structure of human lysozyme. *Proc. Natl. Acad. Sci. U.S.A.* **2007,** *104*, 4846-4851.
5. W. Kabsch, XDS. *Acta Cryst.* **2010***, D66*, 125-132.
6. P. D. Adams, P. V. Afonine, G. Bunkoczi, V. B. Chen, I. W. Davis, N. Echols, J. J. Headd, L. W. Hung, G. J. Kapral, R. W. Grosse-Kunstleve, A. J. McCoy, N. W. Moriarty, R. Oeffner, R. J. Read, D. C. Richardson, J. S. Richardson, T. C. Terwilliger, P. H. Zwart, PHENIX: a comprehensive Python-based system for macromolecular structure solution. *Acta Crystallogr. Sect. D* **2010,** *66*, 213-221.
7. A. J. Mccoy, R. W. Grosse-Kunstleve, P. D. Adams, M. D. Winn, L. C. Storoni, R. J. Read, Phaser crystallographic software. *J. Appl. Crystallogr.* **2007,** *40*, 658-674.
